# Establishment of intestinal organoid cultures modeling injury-associated epithelial regeneration

**DOI:** 10.1038/s41422-020-00453-x

**Published:** 2021-01-08

**Authors:** Molong Qu, Liang Xiong, Yulin Lyu, Xiannian Zhang, Jie Shen, Jingyang Guan, Peiyuan Chai, Zhongqing Lin, Boyao Nie, Cheng Li, Jun Xu, Hongkui Deng

**Affiliations:** 1 School of Basic Medical Sciences, State Key Laboratory of Natural and Biomimetic Drugs, Peking University Health Science Center and the MOE Key Laboratory of Cell Proliferation and Differentiation, College of Life Sciences, Peking-Tsinghua Center for Life Sciences, Peking University, Beijing, 100191 China; 2 School of Life Sciences, Center for Bioinformatics, Center for Statistical Science, Peking University, Beijing, 100871 China; 3 Department of Neurobiology, Capital Medical University, Beijing, 100069 China; 4 Key Laboratory of Cell Proliferation and Differentiation of the Ministry of Education and State Key Laboratory of Membrane Biology, College of Life Sciences, Peking University, Beijing, 100871 China; 5 Beijing Vitalstar Biotechnology Co., Ltd, Beijing, 100000 China; 6 State Key Laboratory of Chemical Oncogenomics, School of Chemical Biology and Biotechnology, Peking University Shenzhen Graduate School, Shenzhen, Guangdong 518055 China

**Keywords:** Intestinal stem cells, Regeneration

## Abstract

The capacity of 3D organoids to mimic physiological tissue organization and functionality has provided an invaluable tool to model development and disease in vitro. However, conventional organoid cultures primarily represent the homeostasis of self-organizing stem cells and their derivatives. Here, we established a novel intestinal organoid culture system composed of 8 components, mainly including VPA, EPZ6438, LDN193189, and R-Spondin 1 conditioned medium, which mimics the gut epithelium regeneration that produces hyperplastic crypts following injury; therefore, these organoids were designated hyperplastic intestinal organoids (Hyper-organoids). Single-cell RNA sequencing identified different regenerative stem cell populations in our Hyper-organoids that shared molecular features with in vivo injury-responsive Lgr5^+^ stem cells or Clu^+^ revival stem cells. Further analysis revealed that VPA and EPZ6438 were indispensable for epigenome reprogramming and regeneration in Hyper-organoids, which functioned through epigenetically regulating YAP signaling. Furthermore, VPA and EPZ6438 synergistically promoted regenerative response in gut upon damage in vivo. In summary, our results demonstrated a new in vitro organoid model to study epithelial regeneration, highlighting the importance of epigenetic reprogramming that pioneers tissue repair.

## Introduction

The derivation of organoids from intestinal tissues revealed a new way to mimic the complex organization and functionality of tissues in vivo.^1^ Remarkably, organoids have been established from multiple adult tissues, such as gut, brain, kidney, lung, and retina, as well as pluripotent stem cells.^2–5^ Because organoids reflect key structural and functional properties of organs, they have been widely used in disease modeling, drug screening, and host–microbe interaction studies.^2,3,6–8^ As a result, organoid technology has become a powerful tool for studying development and diseases in vitro.

One major feature of organoid cultures is the presence of both stem cells and differentiated lineages, which can self-organize into multicellular 3D structures that mimic key histological and functional aspects of the in vivo tissues.^2,8,9^ However, conventional organoid cultures primarily represent homeostasis of stem cells and their derivatives.^1,10–12^ Upon injury in vivo, homeostatic stem cells are generally not sufficient to compensate for the significant loss of tissues.^13–15^ Instead, in tissues with regenerative capacity, such as the skin and gastrointestinal tract, damage generally elicits a regenerative response, especially activation of quiescent reserve stem cells.^16–18^ Increasing evidence indicates that distinct biological programs govern the homeostatic process and injury-associated regeneration, which ensure unnecessary production of stem-progenitors in homeostasis, while maintaining the capacity for highly efficient damage-associated regeneration.^14,15,19^ Modeling the regeneration process in vitro requires cultivation of regenerative tissue with injury-responsive stem cells. Although activation of regeneration is observed during the early stages of organoid formation from single stem cells, this regeneration process is transient which cannot persist during the growth of organoids.^20^

In the gut, for example, regeneration of damaged intestinal epithelium differs significantly in cellular composition and stem cell function from normal intestinal epithelial homeostasis.^15,21–24^ Homeostasis of the intestinal epithelium primarily relies on active cycling of Lgr5^+^ crypt-base columnar cells (CBCs), which give rise to all the major intestinal epithelial cell types.^25,26^ However, upon intestinal injury, the number of Lgr5^+^ CBCs decreases rapidly and dramatically in the gut epithelium, whereas slow cycling reserve intestinal stem cells resistant to DNA damage are activated to repair the injured tissue.^16–18,27–29^ For instance, in a mouse model after irradiation, slow cycling Clu^+^ revival stem cells (revSCs) are activated upon injury and can expand to reconstitute most cell lineages including Lgr5^+^ stem cells to support tissue regeneration.^23^ However, these regenerative responses, particularly the injury-responsive population, have not been captured properly in current intestinal organoid culture, which cannot faithfully mimic the regenerative gut epithelium upon tissue injury in vivo.

Here, we established a culture condition for generating and culturing intestinal organoids with injury-associated regenerative features in vitro. Compared to conventional intestinal organoids, these new organoids showed profound complex crypt–villus structures and significant enrichment of injury-associated regenerative signatures such as fetal-like markers, known as hyperplastic intestinal organoids. Importantly, hyperplastic intestinal organoids significantly enriched injury-responsive stem cell populations similar to those that emerge in vivo upon damage. In this condition, we found that a combination of small-molecule epigenetic modulators VPA and EPZ6438 was critical for regulating hyperplastic features of intestinal organoids, and also promoted regeneration of the gut epithelium upon damage in vivo.

## Results

### Establishment of novel organoid cultures with regenerative features

**Fig. 1:**
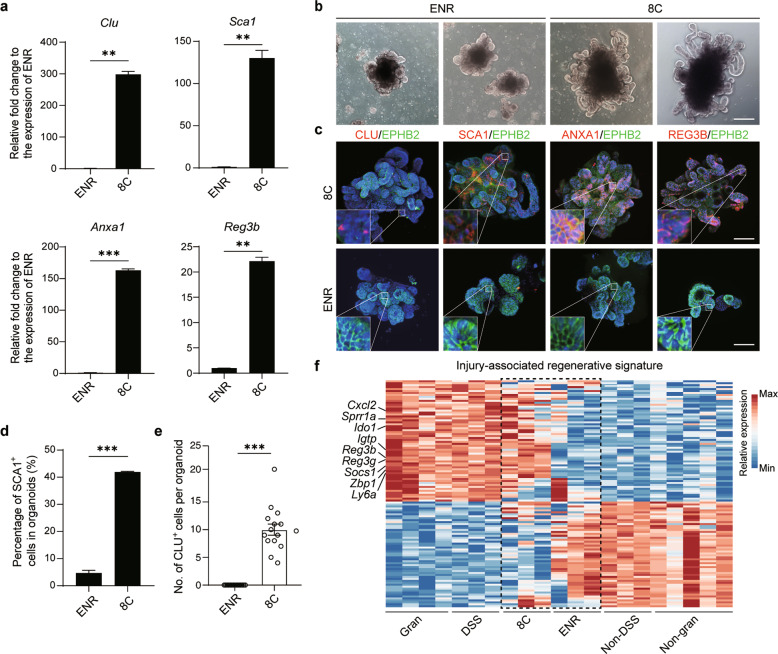
Establishment of intestinal organoid cultures enriched for an injury-associated regenerative signature. **a** qPCR analyses of *Clu* and regenerative gene expression in organoids cultured under the indicated conditions (*n* = 2 wells). *P* values were determined by two-sided unpaired *t*-test. **b** Typical morphology of intestinal organoids cultured under the indicated conditions. **c** Immunofluorescence staining of CLU and regenerative markers in organoids cultured under the indicated conditions. **d** FACS analysis of SCA1^+^ cells in organoids cultured under the indicated conditions (*n* = 3 mice). *P* values were determined by two-sided unpaired *t*-test. **e** Quantification of CLU^+^ cells in each organoid cultured under the indicated conditions (*n* = 15 organoids from three mice). *P* values were determined by two-sided unpaired *t*-test. **f** Heatmap displaying the expression of an injury-associated regenerative signature in different organoids and primary crypts from different intestinal injury models (*n* = 3 mice). The gene expression profile of organoids cultured in the ENR and 8C conditions was plotted. Representative genes are shown on the left. Gran and Non-gran indicate crypts overlying and adjacent to granulomas, respectively. DSS and Non-DSS indicate crypts from repairing epithelium in DSS-associated colitis, and normal epithelium without DSS treatment, respectively. **P < 0.01; ***P < 0.001. Scale bars, 100 μm. The experiments in **a**–**c** were independently repeated at least three times with similar results.

To develop a novel organoid system recapitulating the process of intestinal epithelial regeneration after injury, we focused on identifying factors that support long-term expansion of intestinal organoids expressing an injury-associated regenerative signature. *Clu*, a specific marker of revival stem cells for intestinal regeneration upon damage to intestinal stem cells,^23^ as well as regenerative markers specifically expressed in the injury-associated repairing epithelium,^21–24^ including *Sca1*, *Anxa1* and *Reg3b*, were used as major markers for screening. A panel of cytokines and small molecules targeting signaling and epigenetic pathways that govern stem cell expansion were screened. More than 30 primary hits were found to increase the expression levels of screening markers. Next, different combinations of these candidates were tested to explore their potential to further increase the expression of these markers (Supplementary information, Fig. S1a). To this end, a new culturing condition including 8 components (8C; LDN193189, GSK-3 Inhibitor XV, Pexmetinib, VPA, EPZ6438, EGF, R-Spondin 1 conditioned medium, and bFGF) was found to effectively upregulate the expression of these markers in intestinal organoid cultures (Fig. 1a). Notably, organoids cultured in the 8C condition grew faster than conventional intestinal organoids cultured in the presence of EGF, Noggin and R-Spondin 1 (collectively, the ENR condition^1^) and more robust expansion of Lgr5-GFP^+^ cells was also observed (Supplementary information, Fig. S1b–d). Freshly isolated Lgr5-GFP^+^ cells had a higher organoid-forming efficiency than Lgr5-GFP^–^ cells under the 8C condition (Supplementary information, Fig. S1e–g). Organoids cultured in the 8C condition showed the presence of multiple differentiated intestinal lineages, including goblet (*Muc2*), enteroendocrine (*Chga*), and Paneth (*Lyz1*) cells (Supplementary information, Fig. S1h, i). In addition, organoids cultured in the 8C condition also showed genome stability after more than 20 passages (Supplementary information, Fig. S1j).

Next, we analyzed the regenerative features of organoids in our new culturing medium. Importantly, compared with organoids cultured in the ENR condition, the 8C condition significantly promoted the elongation of budding domains with a larger organoid size and more complex crypt–villus structures, which were morphologically similar to those of the hyperplastic crypts of injury-associated repairing epithelium^21,22,24^ (Fig. 1b; Supplementary information, Fig. S1c). Furthermore, immunofluorescence and flow cytometry analysis showed that key regeneration-associated markers, including SCA1, ANXA1, REG3b, and CLU, were highly expressed in the 8C condition in comparison with the ENR condition (Fig. 1c–e; Supplementary information, Fig. S1k). To functionally examine the regenerative capacity of organoids cultured in the 8C condition, we used an in vitro irradiation model,^30^ and found that transfer of irradiated ENR-cultured organoids into the 8C condition can maintain the growth of organoids without significantly altering the expression of intestinal lineage markers, in contrast to a rapid collapse of ENR-cultured organoids after irradiation (Supplementary information, Fig. S2a–c). These data suggest that organoids cultured in the 8C condition may acquire the regenerative features of in vivo injury-associated hyperplastic intestinal epithelium.

To further explore the regenerative features of organoids from the 8C condition at the transcriptome level, we performed RNA sequencing (RNA-seq) analysis to examine the expression of regeneration-associated genes. Gene set enrichment analysis (GSEA) showed that reported regeneration-associated genes, including those involved in YAP signaling and a fetal intestinal signature, were significantly enriched in the 8C condition in comparison with the ENR condition (Supplementary information, Fig. S3a). In addition, a previously reported colitis-associated regenerative epithelial signature^22^ was also highly enriched in the 8C condition (Supplementary information, Fig. S3a). To further verify the hyperplastic signature of organoids, we defined a comprehensive injury-associated regenerative signature underlying regeneration of the repairing epithelium after injury induced by irradiation,^23^ parasitic helminth infection^21^ and dextran sulfate sodium (DSS) treatment^24^ (Supplementary information, Table S1). Importantly, the 8C condition was associated with significant enrichment of the injury-associated regenerative signature, which resembled the phenotype of hyperplastic crypts upon damage in vivo (Fig. 1f; Supplementary information, Fig. S3b). Consistent with this result, correlation and dimension reduction analysis of the expression of injury-associated regenerative signature genes revealed that organoids cultured in the 8C condition resembled hyperplastic crypts from injury-associated repairing epithelium in different injury models more closely than those cultured in the ENR condition (Supplementary information, Fig. S3c, d). Collectively, these results indicate that organoids cultured in the 8C condition have a hyperplastic phenotype mimicking that of hyperplastic crypts upon damage in vivo.^21,24,31^ Therefore, we designated these organoids as hyperplastic intestinal organoids (Hyper-organoids).

### scRNA-seq analysis of hyper-organoids revealed their unique lineage composition

**Fig. 2:**
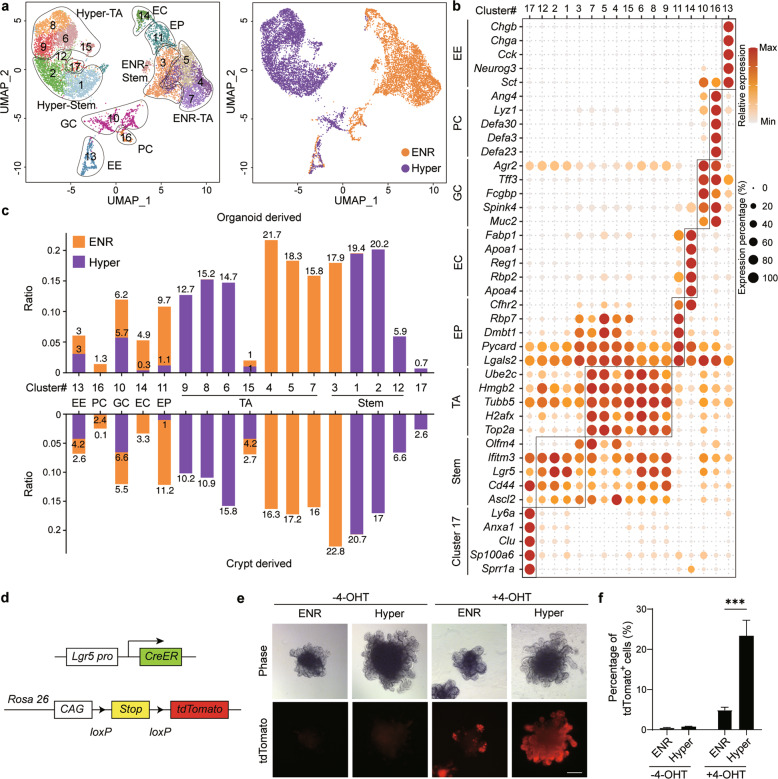
The lineage composition of hyperplastic intestinal organoids resembles that of primary crypts upon damage in vivo. **a** UMAP visualizations of scRNA-seq data from Hyper- and ENR-organoids. Left, colors indicate the unsupervised clusters. Right, colors indicate different organoids: ENR-organoids (orange dots) and Hyper-organoids (purple dots). **b** Expression of cell type-specific markers that identify distinct cell types. EE enteroendocrine, PC Paneth cell, GC goblet cell, EC enterocyte, EP enterocyte progenitor, TA transit amplifying. **c** Stacked histograms showing the proportions of the included cell types in Hyper-organoids derived from either ENR-organoids (up) or primary crypts (down). Hyper-organoids derived from ENR-organoids had a lineage composition similar to those derived from primary crypts. **d** Schematic diagram of the genetic lineage tracing strategy used to trace endogenous Lgr5 activation. **e** Representative images of ENR- and Hyper-organoids (with or without 4-OHT induction) using the lineage tracing system. Scale bars, 100 μm. **f** FACS analysis of tdTomato^+^ cells in ENR- and Hyper-organoids (with or without 4-OHT induction) (*n* = 3 wells). *P* values were determined using one-way ANOVA. ***P < 0.001. Experiments in **e** and **f** were independently repeated at least twice with similar results.

Next, we analyzed the lineage composition of Hyper-organoids using single-cell RNA-seq (scRNA-seq), with organoids cultured in the ENR condition used as controls. Unsupervised clustering of the merged datasets across different organoids revealed 17 distinct epithelial clusters (Fig. 2a, b; Supplementary information, Fig. S4a). In comparison with ENR-organoids, Hyper-organoids showed significant reductions in the abundance of Paneth cells (PCs) and enterocytes, as well as a slight increase in the number of enteroendocrine cells, similar to injury-associated lineage dynamics observed in vivo^21,22^ (Fig. 2c; Supplementary information, Fig. S4b).

Using reported intestinal stem cell markers, clusters 1, 2, and 12 from the Hyper-organoids, as well as cluster 3 from the ENR-organoids, were identified as Lgr5^+^ intestinal stem cells (Fig. 2a–c; Supplementary information, Fig. S4a). Notably, despite shared expression of intestinal stem cell markers (*Lgr5*, *Cd44*, and *Ascl2*), there was a clear separation of the Lgr5^+^ stem cell populations as well as transit amplifying (TA) cells between Hyper-organoids and ENR-organoids (Fig. 2a; Supplementary information, Fig. S4c). To determine the differences of Lgr5^+^ stem cells between Hyper- and ENR-organoids, we integrated the sequencing data of Lgr5^+^ stem cells from different types of organoids as well as homeostatic and injury-associated intestinal epithelium^21,23^ (Supplementary information, Fig. S5a). We found that there were Lgr5-high and Lgr5-low subsets in ENR-organoids, while Lgr5-low subset rarely appeared in Hyper-organoids (Supplementary information, Fig. S5e). Although Lgr5^+^ population in the Hyper-organoids showed closer transcriptomic relationship with Lgr5-low subset from the ENR-organoids when compared to Lgr5-high subset in the ENR-organoids, the global transcriptomic features of Lgr5^+^ cells in the Hyper-organoids are distinct from that in the ENR-organoids (Supplementary information, Fig. S5f, g). Interestingly, we found that the global gene profiles of Lgr5^+^ stem cells in Hyper-organoids resembled that of Lgr5^+^ stem cells from injury-associated repairing intestinal epithelium (termed as injury-responsive Lgr5^+^ stem cells), and were significantly different from that of Lgr5^+^ stem cells in homeostatic epithelium and ENR-organoids (termed as homeostatic Lgr5^+^ stem cells) (Supplementary information, Fig. S5a–c). Differential gene analysis further showed that injury-responsive Lgr5^+^ stem cells enriched several key regenerative genes, such as *Reg3b*, *Reg3g*, and *Sca1,*^24^ and downregulated the expression of stem cell marker *Olfm4*, which was mainly expressed under homeostatic conditions^32^ (Supplementary information, Fig. S5c, d).

In addition to injury-responsive Lgr5^+^ stem cells, we also found that cluster 17, which contained representative marker genes (*Clu, Anxa1* and *Sca1*) of revival stem cells in an irradiation model,^23^ was uniquely presented in Hyper-organoids (Fig. 2b; Supplementary information, Fig. S4d–f). Notably, the transcriptomic profiles of cluster 17 resembled that of revival stem cells (referred to as SSC2c in in vivo irradiation model^23^) from irradiated crypts (Supplementary information, Fig. S4e). Furthermore, cluster 17 was also enriched in fetal gene signatures, which represent the primitive molecular features of injury-responsive stem cells in vivo (Supplementary information, Fig. S4e, g).^22,23^ Taken together, Hyper-organoids contain Lgr5^+^ and Clu^+^ stem cell populations that resemble the injury responsive stem cells from the gut epithelium upon damage in vivo.

To further investigate the functionality of Lgr5^+^ regenerative stem cells in Hyper-organoids, we first purified Lgr5-GFP^+^ cells from organoids by FACS sorting and tested their self-renewal abilities at the single-cell level. We found that Lgr5^+^ cells from Hyper-organoids had a significantly higher organoid-forming efficiency than those from ENR-organoids (Supplementary information, Fig. S6a). We further traced the progenies of Lgr5-GFP^+^ cells by labeling them with tdTomato expression using a tamoxifen-induced CreERT2 system (Fig. 2d), which showed that progenies of Lgr5^+^ stem cells in Hyper-organoids accounted for more than 20% the total cell population after tamoxifen treatment (Fig. 2e, f; Supplementary information, Fig. S6b). Moreover, the expression of differentiated intestinal markers and regenerative markers co-expressed with tdTomato was also observed (Supplementary information, Fig. S6c, d), suggesting that Lgr5^+^ cells give rise to other progenies in the Hyper-organoids. Directed differentiation of hyper-organoids by modulating the Wnt and Notch signaling pathways significantly increased the expression of differentiated marker genes for different intestinal lineages, including PCs, enterocytes, enteroendocrine cells, and goblet cells (GCs) (Supplementary information, Fig. S6e). Notably, in an in vitro irradiation model, progenies of injury-responsive Lgr5^+^ stem cells showed significant enhanced survival ability whereas those of homeostatic Lgr5^+^ stem cells died completely (Supplementary information, Fig. S2d, e). Collectively, these data suggested that Hyper-organoids contained functional Lgr5^+^ regenerative stem cells.

### VPA and EPZ6438 were critical for regulating hyperplastic features of intestinal organoids

**Fig. 3:**
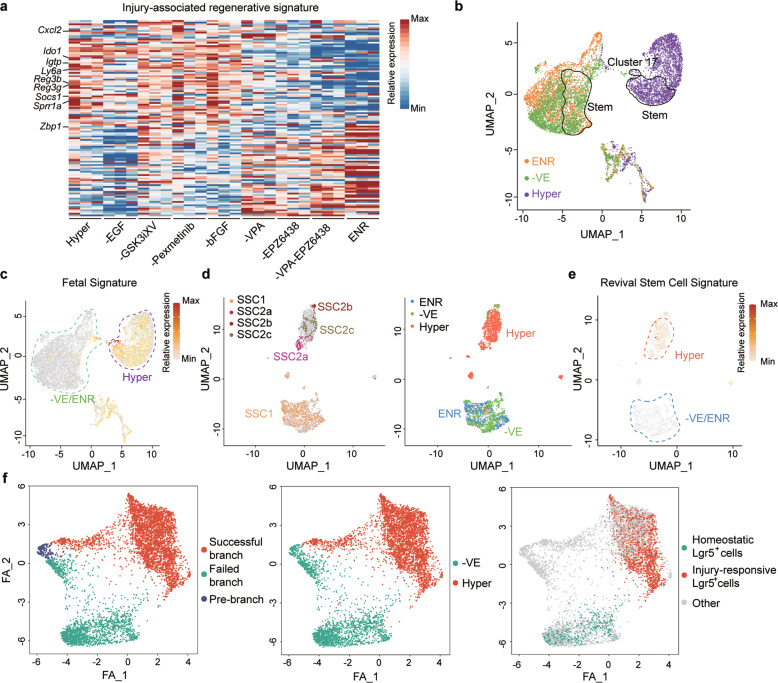
VPA and EPZ6438 are critical for establishing a hyperplastic phenotype in intestinal organoids. **a** The effects of individual components of the 8C medium on the expression of an injury-associated regenerative signature in Hyper-organoids (*n* = 3 mice). Representative genes are shown on the left. **b** UMAP visualizations of scRNA-seq data across different organoids. The stem cell clusters and cluster 17 annotated in Fig. 2a are plotted. Colors indicate different organoids: ENR-organoids (orange dots), Hyper-organoids (purple dots), and organoids cultured in 8C minus VPA/EPZ6438 condition (–VE) (green dots). **c** Expression of the fetal gene signature was overlaid on the UMAP shown in **b**. Hyper- and –VE/ENR-organoids are separated by the purple and green dotted line. **d** Integrated analysis of stem cell clusters from different organoids in vitro and the irradiation model in vivo. **e** Expression of the revival stem cell signature was overlaid on the UMAP shown in **d**. Hyper- and –VE/ENR-organoids are separated by the orange and blue dotted line. **f** Trajectory reconstruction of single cells from Hyper-organoids with and without VPA/EPZ6438 treatment. Left panel: pre-branch (blue dots, before bifurcation), successful branch (red dots), and failed branch (green dots). Middle panel: cells from the Hyper-organoids (red dots) and –VE-organoids (green dots) were overlaid on the regenerative trajectory, respectively. Right panel: distribution of homeostatic (green dots), injury-responsive (red dots) Lgr5^+^ cells, and other cells (gray dots) were overlaid on the regenerative trajectory.

To mechanistically explore the acquirement of regenerative features in Hyper-organoids, we first analyzed the effect of removing individual factors from the 8C condition. Removal of LDN193189 and R-Spondin 1 conditioned medium resulted in growth retardation of organoids. EGF and GSK3iXV were necessary for the continuous passage of organoids, and the addition of bFGF was beneficial for organoid growth (data not shown). Notably, we also found that GSK3iXV, Pexmetinib, VPA, and EPZ6438 were required to maintain the expression of regenerative markers after passaging (Supplementary information, Fig. S7a). Importantly, simultaneous removal of VPA and EPZ6438 led to rapidly and significantly decreased injury-associated regenerative signatures in the organoid within 7 days, whereas omission of GSK3iXV or Pexmetinib did not have this effect (Fig. 3a). Accordingly, VPA and EPZ6438 could be the major drivers of regenerative features in the Hyper-organoids.

We further performed GSEA analysis to analyze the relationship between regenerative features and VPA/EPZ6438 treatment. Downregulation of the reported regeneration-associated signatures in Hyper-organoids was observed upon the removal of VPA and EPZ6438 (Supplementary information, Fig. S7b). In addition, removal of VPA and EPZ6438 also significantly reduced the percentage of fetal gene-expressing cells, and downregulated fetal gene expression (Fig. 3c; Supplementary information, Fig. S7b, f), suggesting that VPA and EPZ6438 are required by Hyper-organoids to maintain a primitive state. Moreover, the dependence of the expression of injury-associated regenerative signature on these two compounds was also supported by dimension reduction analysis, which showed that removal of VPA and EPZ6438 made Hyper-organoids more closely resemble crypts from the homeostatic epithelium (Supplementary information, Fig. S7c). Consistent with these analyses, organoids cultured in the 8C minus VPA/ERZ6438 condition showed reduced complexity of crypt–villus structures and gradually lost Lgr5-GFP expression during serial passages (Supplementary information, Fig. S7d–f).

To further investigate the time window of the induction of the regeneration signature, we performed transcriptional analysis of organoids at different time points during the early stage of converting ENR-organoids into the 8C condition with and without VPA/EPZ6438. We found that representative genes of the regeneration signature were upregulated at day 2 after transferring ENR-organoids to the 8C condition, and were further increased from day 6 to day 8 (Supplementary information, Fig. S7g). In contrast, the upregulation of regenerative markers was significantly reduced upon the removal of VPA/EPZ6438 (Supplementary information, Fig. S7g), suggesting that these two small molecules induced the regeneration signatures at the early stage of 8C treatment. Importantly, we found that addition of VPA and EPZ6438 in ENR condition was sufficient to promote hyperplastic phenotypes of intestinal organoids (Supplementary information, Fig. S8a–d). Collectively, these results suggest that VPA and EPZ6438 are important for regulating the regenerative features of Hyper-organoids.

Next, we examined the effects of VPA and EPZ6438 on the stem cell composition of Hyper-organoids. Unsupervised clustering showed that Hyper-organoids without VPA/EPZ6438 treatment were transcriptomically clustered together with ENR-organoids, especially the stem cell clusters (Fig. 3b, d). In addition, removal of VPA/EPZ6438 led to the conversion of injury-responsive Lgr5^+^ stem cells back to homeostatic Lgr5^+^ stem cells (Fig. 3b, d), and also significantly decreased the expression of the revival stem cell signature in Hyper-organoids (Fig. 3e). These results reinforce the notion that VPA and EPZ6438 are critical for the development of injury-responsive stem cell populations in Hyper-organoids.

To further investigate the effect of VPA/EPZ6438 treatment on the induction of injury-responsive stem cells in Hyper-organoids, we reconstructed the regenerative trajectory by performing single-cell analysis at different time points in Hyper-organoids with and without VPA/EPZ6438. Interestingly, cells from Hyper-organoids were mainly enriched along the route starting from PCs, in which GCs and TA cells progressed toward Lgr5^+^ stem cells (Supplementary information, Fig. S9a). Notably, trajectory reconstruction of PC, GC, TA, Lgr5^+^ stem cells, and revSC-like cells from Hyper-organoids with and without VPA/EPZ6438 revealed two diverse branches (Fig. 3f). VPA/EPZ6438 treatment drove one branch to form injury-responsive Lgr5^+^ stem cells and Clu^+^ revival-like stem cell population, representing the successful branch (Fig. 3f; Supplementary information, Fig. S9a, c). Interestingly, we found that co-expression of PC marker *Lyz1* and stem cell markers were dominant in the early pseudo-stage of the route, suggesting that regenerative stem cells potentially originated from PCs in the process of regeneration (Supplementary information, Fig. S9a, b, d). Collectively, our findings support a VPA/EPZ6438-mediated regenerative trajectory towards the generation of injury-responsive stem cells in Hyper-organoids.

### Regulation of YAP signaling is important for regenerative responses driven by VPA and EPZ6438 in hyper-organoids

**Fig. 4:**
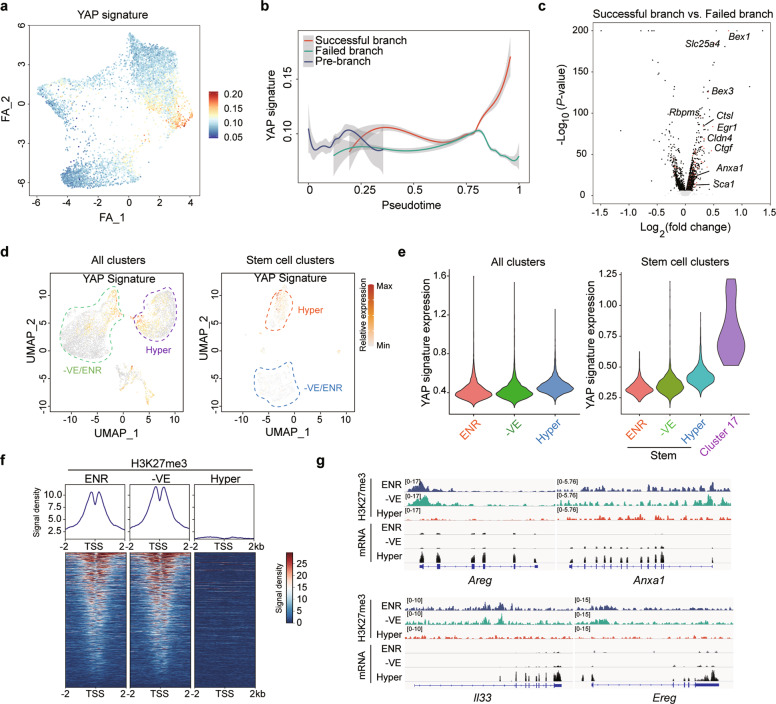
Epigenetic regulation of YAP is involved in the VPA-and-EPZ6438-driven regenerative response in vitro. **a** Expression of the YAP gene signature was overlaid on the regenerative trajectory from Hyper-organoids with and without VPA/EPZ6438 treatment shown in Fig. 3f. **b** The expression dynamics of YAP signature was cataloged in a pseudotime manner shown as a red line (successful reprogramming), a green line (failed reprogramming) and a blue line (pre-branch before bifurcation). Thick lines indicate the average gene expression patterns in each branch. **c** Volcano plot displaying the results of differential gene expression analysis performed between successful branch and failed branch. The dots representing YAP target genes are indicated as red. **d** Expression of the YAP gene signature was overlaid on the UMAPs shown in Fig. 3b and d, respectively. Hyper- and –VE/ENR-organoids are separated by the orange and blue dotted line. **e** Violin plots showing the entire range of metagene expression levels per single cell per cluster for the transcriptional programs of all clusters (left) and stem cell clusters (right) in different organoids. **f** Heatmap displaying H3K27me3 signals at promoter regions of YAP target genes in different organoids. **g** ChIP-seq (H3K27me3) and RNA-seq tracks of YAP target genes in different organoids.

VPA and EPZ6438 are reported to epigenetically regulate global histone modifications,^33,34^ and we indeed found that their combination led to globally reduced H3K27 trimethylation and upregulated H3K27 acetylation in Hyper-organoids (Supplementary information, Fig. S10a). To further investigate the effects of these epigenetic changes on transcriptional regulation of regeneration in vitro, we analyzed differential gene expression between Hyper-organoids and those cultured under the 8C minus VPA/EPZ6438 condition. Notably, genes that are targets of YAP signaling were upregulated in Hyper-organoids (Supplementary information, Fig. S10b, c). In consistent with these observations, we also found reduced levels of H3K27me3 at YAP target genes in Hyper-organoids as compared to that in organoids cultured in the absence of VPA/EPZ6438 (Fig. 4f, g), revealing a potential epigenetic mechanism underlying YAP-dependent intestinal regeneration.

We further explored the roles of Yap signaling in VPA/EPZ6438-driven regenerative response using single-cell sequencing data. Notably, we observed enrichment of YAP signaling along the regenerative trajectory of Hyper-organoids, which was absent in the failed branch without VPA/EPZ6438 treatment (Fig. 4a–c). Consistent with this observation, the upregulation of the YAP transcriptional program in Hyper-organoids, especially in stem cell clusters, was dependent on the addition of VPA and EPZ6438 (Fig. 4d, e). Using SCENIC, we further analyzed the effects of VPA/EPZ6438 treatment on the gene regulatory network in Hyper-organoids. By comparing Hyper-organoids with ENR-organoids and Hyper-organoids without VPA/EPZ6438 treatment, we identified the gene regulatory network specifically active in Hyper-organoids, which includes one top transcriptional factor Tead2 that can form complex with YAP/TAZ^19^ (Supplementary information, Fig. S11a). Importantly, the enrichment of Tead2 regulon was observed in Hyper-organoids, especially in injury-responsive Lgr5^+^ stem cells, which was dependent on the treatment of VPA and EPZ6438 (Supplementary information, Fig. S11b–d). Moreover, organoid-forming efficiency was significantly impaired by the knockdown of *Tead2* in Hyper-organoids (Supplementary information, Fig. S11e), suggesting the requirement of Tead2 regulon in maintaining the self-renewal ability of Hyper-organoids. Collectively, these data suggest that Yap signaling is important for regulating the molecular features of regeneration in Hyper-organoids, which is driven by VPA/EPZ6438 treatment.

YAP signaling is crucial for intestinal regeneration.^23,32^ Therefore, we examined whether YAP also regulates regenerative responses in Hyper-organoids. We observed a marked arrest of Hyper-organoid growth after exposure to Verteporfin (VP), a potent inhibitor of YAP^35^ (Supplementary information, Fig. S10d). Moreover, the addition of LPA, a YAP signaling agonist that functions by inhibiting LATS1/2,^36^ partially compensated for the absence of VPA and EPZ6438 in establishing regenerative features in Hyper-organoids (Supplementary information, Fig. S10d).

Interestingly, in addition to YAP signaling, the Wnt signaling pathway also showed significant enrichment in the successful branch during the regenerative process as indicated by KEGG analysis (Supplementary information, Fig. S10e). Moreover, the expression of regenerative markers was significantly reduced in Hyper-organoids after treatment with Wnt/β-catenin signaling inhibitor XAV939 (Supplementary information, Fig. S10f). Therefore, other signaling pathways like Wnt could also be important for driving the regenerative responses in Hyper-organoids.

### VPA and EPZ6438 promote intestinal and colonic regeneration following damage in vivo

**Fig. 5:**
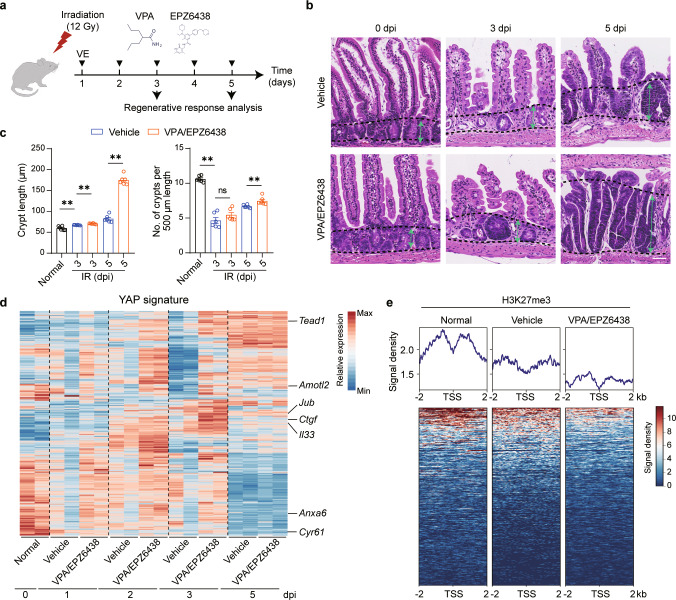
Combined treatment with VPA and EPZ6438 promotes regenerative responses in the intestinal epithelium after irradiation. **a** Schematic diagram of the administration of VPA/EPZ6438 to C57BL/6 mice in the irradiation-induced injury model. **b** H&E staining of small intestinal crypts at 3 and 5 days post-irradiation (dpi). The green arrows between the two dashed lines show the length of the crypts. Scale bars, 50 μm. **c** Quantification of crypt number (10 images were analyzed per mouse) and crypt length (10 crypt–villus axes were counted per mouse) at 3 and 5 dpi (*n* = 6 mice). *P* values were determined by two-tailed Mann–Whitney test. **d** Heatmap displaying the expression of the YAP signature in small intestinal crypts with or without VPA/EPZ6438 treatment at 3 dpi. Representative genes are shown on the right. (*n* = 2 mice). **e** Heatmap displaying H3K27me3 signals at promoter regions of YAP target genes in small intestinal crypts at 3 dpi. **P < 0.01; ns, not significant. The experiments in **b**, **c**, and **e** were independently repeated at least twice with similar results.

Since VPA and EPZ6438 were critical for the activation of the injury-associated regenerative signature and phenotype in Hyper-organoids in vitro, we explored whether combined treatment with VPA and EPZ6438 promoted injury-associated intestinal epithelial regeneration in vivo. Following administration of VPA and EPZ6438 in an in vivo irradiation model,^23,32^ we found that the growth of elongated crypts was markedly enhanced, and that the proliferation of crypts was slightly increased (Fig. 5a–c), indicating that combined VPA/EPZ6438 treatment promoted a regenerative response in the intestinal epithelium upon damage in vivo. In support of these in vivo results, when evaluated in an in vitro irradiation model,^37^ irradiated ENR-cultured organoids transferred into the ENR condition with the addition of VPA and EPZ6438 supported the survival of intestinal organoids after irradiation. (Supplementary information, Fig. S8e, f).

To gain more insight into the molecular traits of epithelial regeneration after VPA/EPZ6438 treatment, we analyzed the transcriptome of primary crypts after irradiation. Notably, upregulation of representative marker genes of YAP gene signature was observed in a time-dependent manner after VPA/EPZ6438 treatment (Fig. 5d), suggesting promotion of YAP-dependent intestinal generation in irradiated crypts after VPA/EPZ6438 treatment. Interestingly, we also found a significant downregulation of H3K27me3 enrichment in the Yap target genes after VPA/EPZ6438 treatment (Fig. 5e), suggesting epigenetic regulation of YAP signaling underlines the VPA/EPZ6438-driven regenerative response in vivo. Collectively, these data suggest that combined VPA/EPZ6438 treatment promoted intestinal epithelium regeneration upon injury.

We further investigated the effects of VPA/EPZ6438 treatment in promoting injury-associated epithelial regeneration in a DSS-induced colitis model.^38,39^ We found that combined VPA/EPZ6438 treatment could decrease disease activity index, and attenuate weight loss and colon length reduce (Supplementary information, Fig. S12a–d). In addition, VPA and EPZ6438 treatment markedly reduced pathological changes, with a well-preserved mucosal architecture and small foci of crypt loss, leading to a decreased histological score (Supplementary information, Fig. S12e). Collectively, these data suggest that VPA and EPZ6438 treatment alleviated symptoms of DSS-treated mice.

## Discussion

In this study, we established a hyperplastic intestinal organoid culturing system, which shared regenerative features with hyperplastic intestinal epithelium in vivo. Importantly, by combining our new organoid culturing system with scRNA-seq analysis, we identified unique stem cell populations resembling injury-responsive stem cells that emerged upon damage in vivo in Hyper-organoids. Mechanistic exploration of the regenerative capacity of Hyper-organoids showed that two small-molecule epigenetic modulators VPA and EPZ6438 were critical for the establishment of hyperplastic features, which was dependent on the regulation of YAP signaling. Furthermore, combined treatment with these two small molecules promoted regeneration of the gut epithelium upon damage in vivo.

A major finding of our study is that Hyper-organoids acquired regenerative features similar to hyperplastic crypts from the regenerating intestinal epithelium in vivo. Compared to conventional intestinal organoids, Hyper-organoids have morphological characteristics that more closely resemble those of hyperplastic crypts, including larger organoid size, profoundly complex crypt–villus structures, and high proliferative capacity (Fig. 1b; Supplementary information, Fig. S1b–d). Importantly, these complex 3D morphological characteristics have not been observed in previously reported in vitro models.^22,24^ These features are also supported by transcriptome analysis showing exclusive enrichment of an injury-associated regenerative signature in Hyper-organoids that was transcriptionally comparable to that of injury-induced hyperplastic intestinal epithelium in vivo (Fig. 1f; Supplementary information, Fig. S3a–d). The regenerative capacity of Hyper-organoids was further validated in an in vitro irradiation model (Supplementary information, Fig. S2a–c). This evidence demonstrates that the morphological and molecular phenotype, as well as functionality, of Hyper-organoids reflect the regenerative responses of the gut epithelium upon damage in vivo, demonstrating that Hyper-organoids provide a new in vitro model to explore the process of in vivo intestinal regeneration.

Another notable feature of Hyper-organoids is their robust ability to induce injury-responsive stem cells. Injury-responsive stem cells like reserve stem cells are important for driving the injury-induced regeneration process in vivo.^16–18,27^ In Hyper-organoids, stem cells that resembled injury-responsive Lgr5^+^ stem cells and Clu^+^ revival stem cells were observed (Figs. 1c–e, 3d, e; Supplementary information, Figs. S4d–f, S5a–d). Notably, unsupervised clustering further confirmed the similarity of injury-responsive stem cell populations from Hyper-organoids and primary crypts isolated in vivo (Supplementary information, Figs. S4e, S5a–c). Functional assay further showed that Lgr5^+^ cells from Hyper-organoids have organoid-forming capacity at the single-cell level (Supplementary information, Fig. S6a). Lineage tracing experiment confirmed that Lgr5^+^ cells can give rise to most of differentiated progenies in Hyper-organoids (Supplementary information, Fig. S6c, d). Moreover, similar to the behavior of injury-responsive stem cells in vivo, progenies of Lgr5^+^ stem cells in Hyper-organoids showed significantly enhanced survival ability after irradiation (Supplementary information, Fig. S2d, e). Collectively, the data showed evidence of functional regenerative stem cell populations in Hyper-organoids resembling that emerges during injury-associated regeneration, which is promising for modeling the dynamics of regenerative stem cells in vitro.

Importantly, the combination of two small molecules, VPA and EPZ6438, was critical for the induction of the hyperplastic phenotype of Hyper-organoids. The morphological and molecular features of Hyper-organoids, as well as their injury-responsive stem cell populations, nearly disappeared without treatment with these two small molecules (Fig. 3a–e; Supplementary information, Fig. S7b–f). Moreover, combined treatment with VPA and EPZ6438 in vivo promoted hyperplastic features of the gut epithelium after irradiation, confirming the functionality of VPA and EPZ6438 in contributing to the hyperplastic phenotype (Fig. 5a–c). Interestingly, VPA and EPZ6438 are required for the development of a primitive state in Hyper-organoids, as we found nearly complete loss of fetal gene-expressing cells without treatment with these two small molecules (Fig. 3c; Supplementary information, Fig. S7f). Previous findings support the notion that injury induces dedifferentiation of intestinal lineages to a more primitive state in vivo.^40–44^ By reconstructing the regenerative trajectory, we observed the starting cell population majorly contained PCs, which progressed toward Lgr5^+^ stem cells before bifurcation, dependent on the treatment of VPA and EPZ6438 (Fig. 3f; Supplementary information, Fig. S9). This observation was consistent with a significant reduction in the abundance of these PCs in Hyper-organoids (Fig. 2c; Supplementary information, Fig. S4b), supporting previous reports that dedifferentiation of intestinal terminal lineages could contribute to intestinal stem cell restoration.^44,45^ Thus, our findings support the presence of a VPA-and-EPZ6438-induced dedifferentiation mechanism underlying the generation of injury-responsive stem cells in Hyper-organoids, which merits further investigation.

Finally, our study also revealed the importance of the YAP signaling pathway in maintaining the regenerative features of Hyper-organoids. YAP target genes were activated by combined treatment with VPA/EPZ6438 (Supplementary information, Fig. S10b, c). Further analysis of the regulatory network in Hyper-organoids showed that VPA/EPZ6438 were important for regulating Tead2 (Supplementary information, Fig. S11a), which is one top transcription factor in the regulatory network of Hyper-organoids and a major downstream effector of YAP signaling.^19^ We further manipulated the activity of YAP signaling in Hyper-organoids, and the results showed that inhibition of YAP signaling greatly disturbed VPA/EPZ6438-induced regenerative features whereas YAP activation could partially compensate for the roles of VPA/EPZ6438 in eliciting regenerative response in Hyper-organoids (Supplementary information, Fig. S10d). Interestingly, H3K27me3 levels in the locus of YAP target genes were significantly reduced by VPA/EPZ6438 treatment (Fig. 4f, g), which provides novel insight into the YAP-dependent mechanisms underlying the intestinal regeneration process. Collectively, these results suggested that YAP activation played an important role in regulating the regenerative response in Hyper-organoids. In addition to YAP signaling, other signalings like Wnt could also be critical for regulating hyperplastic features in Hyper-organoids. Recent reports demonstrated a critical role of RXR signaling in the regulation of the regeneration state in intestinal organoids.^20^ Interestingly, compared to organoids cultured in the 8C minus VPA/EPZ6438 condition, Hyper-organoids showed a slightly decreased expression of target genes of RXR signaling (Supplementary information, Fig. S10g, h).

In summary, our study provides a novel platform for modeling injury-induced intestinal regeneration in vitro, which can be utilized to perform screening to identify candidates that could promote intestinal regeneration, as well as to reveal novel insights into mechanisms of intestinal regeneration. Moreover, our approach can be adopted to establish in vitro 3D regeneration models for other regenerative stem cells in different tissues and organs in the future.

## Materials and methods

### Mice

All animal procedures were performed according to NIH guidelines. All practices related to mice were approved by the Institutional Animal Care and Use Committee of Peking University. *Lgr5-EGFP-IRES-creERT2* mice were kindly provided by Ting Chen (National Institute of Biological Sciences), *Rosa26-loxp-STOP-loxp-tdTomato* mice were obtained from Jackson Labs (Stock No. 007914), and C57BL/6 mice were purchased from Charles River (Beijing). The *Lgr5-EGFP-IRES-creERT2* mice were crossed with *Rosa26-lox-STOP-lox-tdTomato* mice to prepare crypt cells for tracing *Lgr5*. 6- to 12-week-old mice were used for crypt isolation and organoid culture. 8-week-old female mice were used for irradiation experiments and generation of the experimental model of colitis.

### Crypt isolation and organoid culture

Crypts were isolated and cultured as previously described.^1^ Briefly, proximal small intestine sections of approximately 20 cm were harvested, opened longitudinally, cut into 2–4 mm pieces, rinsed with cold PBS and incubated in 2 mM EDTA at 4 °C for 30 min with gentle shaking. Crypts were released after vigorous shaking in cold PBS and passed through a 70 μM cell strainer (BD Biosciences) for further enrichment. Approximately 500 crypts were embedded in 20 μL Matrigel (growth factor reduced; BD bioscience) and plated in 48-well plates. After Matrigel polymerization, 500 μL culture medium containing Advanced DMEM/F12 (Gibco), 10 mM HEPES (Gibco), 1× GlutaMAX (Gibco), 1% penicillin/streptomycin (Gibco), 1× N2 Supplement (Gibco), 1× B27 Supplement (Gibco), and 1 mM N-Acetylcysteine (Sigma-Aldrich) was added. For ENR-organoids, culture medium containing 50 ng/mL murine EGF, 100 ng/mL murine Noggin and 500 ng/mL human R-Spondin 1 was used. For Hyper-organoids, culture medium containing 0.2 μM LDN193189, 0.1 μM GSK-3 Inhibitor XV, 1 μM Pexmetinib, 500 μM VPA, 2 μM EPZ6438, 50 ng/mL murine EGF, 10% R-Spondin 1 Conditioned Medium (Trevigen), and 20 ng/mL FGF2 was used. The cell culture medium was changed every other day. Hyper-organoids can be generated from either ENR-organoids or primary crypts. For passage, organoids were removed from Matrigel, mechanically dissociated into single-crypt domains and transferred to fresh Matrigel. Passage was performed every 7 days with a 1:8 split ratio for ENR-organoids and a 1:30 split ratio for Hyper-organoids. 5% knockout serum replacement (KSR, GIBCO) was recommended to enhance expansion of Hyper-organoids after they were passaged. Six days after passage, the number of colonies containing typical organoid structures and expressing Lgr5-GFP was counted.

Directed differentiation of organoid cultures was performed as previously reported.^46^ In brief, ENR- and Hyper-organoids were cultured under ENR conditions, supplemented with Notch inhibitor DAPT (10 μM) to induce enterocyte (*alpi*); with Notch inhibitor DAPT (10 μM) and Wnt inhibitor IWP2 (2 μM) to induce PC (*lyz1*), GC (muc2) and enteroendocrine cell (*chga*).

### Quantitative PCR analysis

The total RNA from an entire well of cultured cells was isolated using the RNeasy Mini Kit (QIAGEN, 74106). RNA was converted to cDNA using Trans-Script First-Strand cDNA Synthesis SuperMix (TransGen Biotech). Quantitative PCR (qPCR) was performed using KAPA SYBR FAST qPCR Kit Master Mix (KAPA Biosystems) on a CFX Connect^TM^ Real-Time System (Bio-Rad). The data were analyzed using the delta-delta Ct method. β-actin was used as a control to normalize the expression of target genes. The primer sequences used for qPCR in this study are listed in Supplementary information, Table S2.

### Immunofluorescence staining

Cultured cells were fixed in 4% paraformaldehyde (DingGuo) at room temperature for 15 min and permeabilized with PBS–0.1% Triton X-100 (Sigma-Aldrich). The cells were incubated with primary antibodies overnight at 4 °C, followed by incubation with secondary antibodies at 37 °C for 1 h. The nuclei were stained with DAPI (Roche Life Science).

### Irradiation-associated injury model in vitro

For the in vitro irradiation-associated damage experiments, the cultured organoids were exposed to a single dose of 6 Gy irradiation 24 h after seeding using a GammaCell 40 irradiator. After irradiation, irradiated organoids were immediately transferred to an incubator for organoid culture.

### Flow cytometry analysis

Cell culture medium was removed and TrypLE Express (Gibco) was added. After incubation at 37 °C for 10 min, organoids were dissociated into single cells, stained with Sca1-PECy5 at 4 °C for 30 min, and washed three times with PBS. The cells were then stained with 7-aminoactinomycin D (7-AAD) and filtered through a 40 μm cell strainer. Finally, the cells were analyzed on a BD LSRFortessa machine. Data analysis was performed using FlowJo software (Ashland).

### Identification of injury-associated regenerative signature

Published datasets from different intestinal injury models were filtered, and differentially expressed genes between biopsies of injured and non-injured crypts were analyzed. In a parasitic helminth infection model^21^ (GSE97405), 1101 upregulated genes (FDR ≤ 0.05, fold-change comparison of > 1) and 564 downregulated genes (FDR ≤ 0.05, fold-change comparison of > 1.4) were identified. In the DSS model^24^ (E-MTAB-5249), 681 upregulated genes (FDR ≤ 0.05, fold-change comparison of > 1) and 1444 downregulated genes (FDR ≤ 0.05, fold-change comparison of > 1) were identified. In an irradiation model^23^ (GSE117783), 1673 upregulated genes (FDR ≤ 0.05, fold-change comparison of > 1) and 1002 downregulated genes (FDR ≤ 0.05, fold-change comparison of > 1) were identified. The injury-associated regenerative signature, including 63 upregulated genes and 54 downregulated genes, was generated by determining the overlapped changes of these three injury conditions.

Differential gene analysis was carried out for the injury-responsive Lgr5^+^ and homeostasis Lgr5^+^ cells in DSS-induced colitis and upon irradiation or Parasitic helminth infection. The genes enriched in injury-responsive Lgr5^+^ cells in at least two of the above intestinal injury models were defined as injury-responsive Lgr5 signature, with a total of 768 genes. Genes enriched in homeostatic Lgr5^+^ cells in at least two of the above intestinal injury models are defined as homeostatic Lgr5 signature, with a total of 1057 genes.

### Bulk RNA-seq

Total RNA was isolated using the RNeasy Mini Kit (QIAGEN). RNA sequencing libraries were constructed using the NEBNext^®^ Ultra RNA Library Prep Kit for Illumina^®^ (NEB England BioLabs). Fragmented and randomly primed 2 × 150 bp paired-end libraries were sequenced using an Illumina HiSeq X Ten system. Functional enrichment of previously reported and newly defined gene sets in the transcriptomes across different organoids was determined using the GSEA software package.

### scRNA-seq

The sampling details for the scRNA-seq experiments are shown in Supplementary information, Table S3. To isolate single cells, Hyper-organoids and ENR-organoids were incubated with TrypLE Express at 37 °C for 20 min, after which live cells were counted using a hemocytometer. On average, ~3000 captured individual cells per sample were subjected to 10× Genomics single-cell isolation and RNA-seq following the manufacturer’s recommendations. Using single cell 3′ Library and Gel Bead Kit V3 (10× Genomics, 1000075) and Chromium Single Cell B Chip Kit (10× Genomics, 1000074), the cell suspension (300–600 living cells per microliter determined by Count Star) was loaded onto the Chromium single cell controller (10× Genomics) to generate single-cell gel beads in the emulsion according to the manufacturer’s protocol. In short, single cells were suspended in PBS containing 0.04% BSA. Captured cells were lysed and the released RNA was barcoded through reverse transcription in individual GEMs. Reverse transcription was performed on a S1000TM Touch Thermal Cycler (Bio Rad) at 53 °C for 45 min, followed by 85 °C for 5 min, and hold at 4 °C. The cDNA was generated and then amplified, and quality assessed using an Agilent 4200 (performed by CapitalBio Technology, Beijing). According to the manufacture’s introduction, scRNA-seq libraries were constructed using Single Cell 3′ Library and Gel Bead Kit V3. The libraries were finally sequenced using an Illumina Novaseq6000 sequencer with a sequencing depth of at least 100,000 reads per cell with pair-end 150 bp (PE150) reading strategy (performed by CapitalBio Technology, Beijing).

### Analysis of bulk RNA-Seq data

The raw sequences were cleaned using Trimmomatic (version 0.39), and mapped to mouse reference genome mm10 using STAR (version 2.7.3a). High quality mapped reads were quantified using featureCounts. The raw counts were normalized with further variance stabilization by using DEseq2 for heatmap visualization.

### Analysis of scRNA-seq data

Sequences from 8 scRNA-seq samples were processed with Cellranger (v.3.0.0) software (10× Genomics). The generated filtered cell UMI count matrixes were imported and merged using the Seurat package (version 3.1.4). Cells with fewer than 1500 genes expressed or with more than 25% of reads from mitochondrial genes were discarded. The Scater package (version 3.11) was used for further quality control and deconvolution-based normalization. The normalized data were imported into the Seurat object data slot. Subsequently, highly variable genes (*n* = 2000) were identified; normalized data were scaled and regressed by mitochondrial ratio and total gene counts, and principle component analysis was conducted using default parameters in the Seurat package. A Shared Nearest Neighbor (SNN) network was built according to the top 10 principal components, and cells were clustered using a modularity optimization-based clustering algorithm (Louvain) with resolution of 1.0, which effectively identified most cell types. For each cluster, highly expressed marker genes were identified using Wilcoxon tests and used to manually annotate cell types based on published datasets.^47^ The major cell markers were as follows: stem cell (*Lgr5*, *Ascl2*, *Olfm4*, and *Cd44*); TA cell (*Top2a*, *Ube3c*, *Hmgb2*, and *Tubb5*), PC (*Ang4*, *Lyz1*, *Defa30*, and *Defa3*), enterocyte (*Fabp1*, *Apoa1*, *Reg1*, and *Rbp2*), enterocyte progenitor (*Cfhr2*, *Rbp7*, *Dmbt1*, and *Pycard*), enteroendocrine (*Chgb*, *Chga*, *Cck*, and *Neurog3*), GC (*Agr2*, *Tff3*, *Muc2*, and *Fcgbp*). All unsupervised clusters were readily assigned to a well-established cell type. For the identification of revival stem cell-like cell clusters, which required additional information, a finer resolution (3.0) and additional principal components (*n* = 15) were adopted, and the identified revival stem cell-like cluster was integrated into those of other cell types. The UMAP dimension reduction was calculated based on the top 15 principal components. For the DotPlot, the normalized mean expression level of each gene in each cluster was calculated with the percentage of cells with expression level > 0. The expression level and percentage of each gene were further normalized by the corresponding maximum value within all clusters/groups for each gene. The differentially expressed genes between single cell clusters were identified using the FindMarkers function in Seurat. The significance threshold for the adjusted *P* values was 0.05, and a fold-change ≥ 2 was required. The levels of the revival stem cell^23^ and fetal gene signatures were calculated as the averaged normalized expression level of the corresponding gene sets used in the corresponding related study. The revival stem cell signature included four genes: *Clu*, *Anxa1*, *Cxadr*, and *Basp1*. The fetal gene sets were generated from published datasets.^24^ The YAP signature gene set was a collection of genes that were upregulated > 2 fold in YAP-overexpressing intestinal epithelial cells compared with wild-type ones.^32^

### Integration of bulk datasets from Gran, DSS and hyper organoids models

The raw counts of the Gran (granuloma-associated crypt epithelium in helminth infection model^21^) and Hyper-organoid datasets were log-transformed. Next, these datasets were combined, and only injury-associated regenerative signature genes were used for downstream analysis. Batch effects were corrected using the Combat function in the sva package. Principal component analysis was performed on the scaled merged dataset. The correlation analysis of different models was visualized using a heatmap based on the Spearman correlation coefficients of the log-transformed, batch-corrected, and normalized expression levels of injury-associated regenerative signature genes.

### Gene regulatory network analysis

The analysis of regulon activity in Supplementary information, Fig. S11 was performed by following standard SCENIC pipeline. The input to SCENIC is an expression matrix, in which rows correspond to genes and columns correspond to cells. The cells from ENR-organoids and Hyper-organoids with or without VPA and EPZ6438 treatment were selected as the input cells. The genes with at least 186 UMIs across all cells and detected in at least 62 cells were selected as the input genes. The expression matrix was loaded into GENIE3 and the dataset of co-expressed genes associated with each transcription factor (TF) was constructed. This step is time consuming and was done with high performance computing (HPC). The TF co-expression modules were then analyzed by RcisTarget. The Normalized Enrichment Score (NES) of TF-binding motifs (TFBS) was calculated, and NES > 3.0 was considered as significantly enriched. The filtered potential targets by RcisTarget mouse mm9 database from the co-expression module were used to build the regulons. The regulon activity (Area Under the Curve) was analyzed by AUCell and the active regulons are determined by AUCell default threshold.

### Integration of the dataset of irradiated crypts with organoid scRNA-seq dataset

The 10× sequencing dataset of irradiated crypts and normal control sample dataset were downloaded from the Gene Expression Omnibus (GEO) (GSE123516) and merged. Cell types were clustered and annotated manually using the pipeline described above. Specifically, CBCs from the irradiated crypt dataset were classified into SSC1 and SSC2a/b/c subtypes. As we were interested in stem cells, the raw counts for stem cell clusters from irradiated crypts, as well as the ENR- and Hyper-organoid datasets, were merged. The combined dataset was further normalized, scaled, and regressed, after which principal component analysis was conducted as previously described. The batch effect between the irradiated crypt and organoid datasets was corrected using Harmony (version 1.0). The downstream graph-based clustering and UMAP analysis were based on the top 15 adjusted Harmony coordinates. To compare the stem cell clusters in Hyper-organoids cultured in the presence or absence of VPA/EPZ6438, the raw counts of Hyper-organoids without VPA/EPZ6438 treatment were added and subject to the downstream analysis. For visualization, only cells from irradiated crypts, S61, S62, and S63 were plotted (the sample information of S61, S62 and S63 is shown in Supplementary information, Table S3).

### Single-cell trajectory analysis

Single-cell trajectory in Fig. 3f and Supplementary information, Fig. S9a was reconstructed by RNA velocity and R package phateR, respectively. RNA velocity-based cell fate tracing was performed using scVelo python module. The spliced reads and unspliced reads were recounted by the velocyto python module based on previous aligned bam files of cellranger output. The velocities were calculated using scvelo.tl.velocity function with default parameters. The transition probabilities are computed using cosine correlation between the potential cell-to-cell transitions and the velocity vector by scvelo.tl.velocity_graph function. Then the velocity pseudotime which measures the average number of steps it takes to reach a cell after walking along the graph starting from the root cells was calculated based on the velocity graph using scvelo.tl.velocity_pseudotime function. The root cells were implicitly inferred based on the directed velocity graph.

### ChIP and sequencing library preparation

ChIP material was obtained and processed as described previously^48^ with the following modifications. In brief, for each sample, approximately 5 × 10^6^ cells were crosslinked in PBS containing 1% formaldehyde at room temperature for 10 min, after which they were quenched with glycine for 5 min. The fixed cells were washed twice with cold PBS and centrifuged at 500× *g* for 3 min at 4 °C, followed by removal of the supernatant. The cell pellets were re-suspended, and the resulting homogenates were further lysed. Crosslinked material was sonicated using a Covaris sonicator for 12 min at duty 5%, intensity 3, and bursts 200. The size of the sonicated fragments ranged between 400 and 800 bp. Cleared chromatin was incubated with 2.5 mg of a selected antibody overnight at 4 °C. The antibody used in the experiments was anti-H3K27me3 (Cell Signaling Technology, 9733).

### ChIP-seq data analysis

ChIP-seq reads were aligned to mouse genome build mm10 using bwa (v0.7.12)^49^ software. Duplicate PCR reads were removed from the aligned sequences using Picard (version 2.6). Chromatin profiles were calculated over all RefSeq genes and plotted using ngsplot software.

### Western blot analysis

Cells were collected and lysed in RIPA buffer (Beyotime Technology Technology, P0013B) supplemented with protease inhibitor cocktail (Thermo Fisher Scientific, 78443) and phosphatase inhibitor cocktail (Thermo Fisher Scientific, 78428). Samples were resolved by SDS-PAGE and transferred to polyvinylidene difluoride membranes (Millipore), which were then incubated with primary antibodies and HRP-conjugated secondary antibodies (Jackson ImmunoResearch, 111-035-003 and 115-035-003). The membranes were exposed and developed using a film processing machine (Kodak). See Supplementary information, Table S4 for the antibodies used in this study.

### Irradiation-associated intestinal injury and treatments in vivo

Mice were exposed to a single dose of 12 Gy irradiation using a GammaCell 40 irradiator to induce intestinal injury. After irradiation, mice were gavaged daily with VPA (40 mg/kg) and EPZ6438 (0.6 mg/kg). Small intestinal crypts were isolated on different days after irradiation for RNA-seq analysis.

### DSS-associated colitis and treatments

DSS colitis was induced by giving the mice drinking water with 2.5% DSS (molecular mass 36–50 kDa; MP Biomedicals) for 5 days, followed by normal drinking water for the remaining days of the experiment. Mice were injected i.v. daily with VPA (10 mg/kg) and EPZ6438 (0.2 mg/kg), starting from day 5 (DSS withdraw) until they were euthanized. We used the disease activity index (DAI) to quantify colitis severity as previously described.^50^

### Histological analysis

Briefly, total colon was resected and immediately fixed with 10% buffered formalin and embedded in paraffin. 3 μm sections were cut, and stained with hematoxylin and eosin. The histological score of each mouse was calculated according to epithelium damage and cell infiltration.^51^ The specimens were analyzed blindly by a pathologist under a light microscope and the scores were recorded and confirmed as follows: extent of disease (0–3), depth of the lesion (0–3), crypt abscess (0–3), and degree of inflammatory cell infiltration (0–3). The total histologic score was derived by summing each individual score.

### Statistical analysis

All values are depicted as means ± SEM. Statistical parameters, including statistical analysis methods, thresholds for statistical significance, and group sizes are reported in the figure legends and supplementary figure legends. The results for qPCR, FACS and quantification of cells or organoids were evaluated with an unpaired two-tailed Student’s *t*-test. Ki67^+^ crypt number, crypt length, disease activity index, and histological scores were evaluated using a two-tailed Mann–Whitney test. Body weight and colon length were evaluated using one-way ANOVA. Statistical analyses were performed using GraphPad Prism Software (GraphPad).

## Supplementary information

Supplementary Fig. S1

Supplementary Fig. S2

Supplementary Fig. S3

Supplementary Fig. S4

Supplementary Fig. S5

Supplementary Fig. S6

Supplementary Fig. S7

Supplementary Fig. S8

Supplementary Fig. S9

Supplementary Fig. S10

Supplementary Fig. S11

Supplementary Fig. S12

Supplementary Table S1

Supplementary Table S2

Supplementary Table S3

Supplementary Table S4

## Data Availability

The datasets generated in the current study are available in the GEO database. All data are available in the main text or the supplementary materials.
