# Supplementary material for: Establishment of intestinal organoid cultures modeling injury-associated epithelial regeneration
Source: Cell Res. 2021 Jan 8;31(3):259–71. doi: 10.1038/s41422-020-00453-x (PMC8027647; doi:10.1038/s41422-020-00453-x)
Supplement: Supplementary file 1 — Supplementary Fig. S1 [file 41422_2020_453_MOESM1_ESM.pdf]

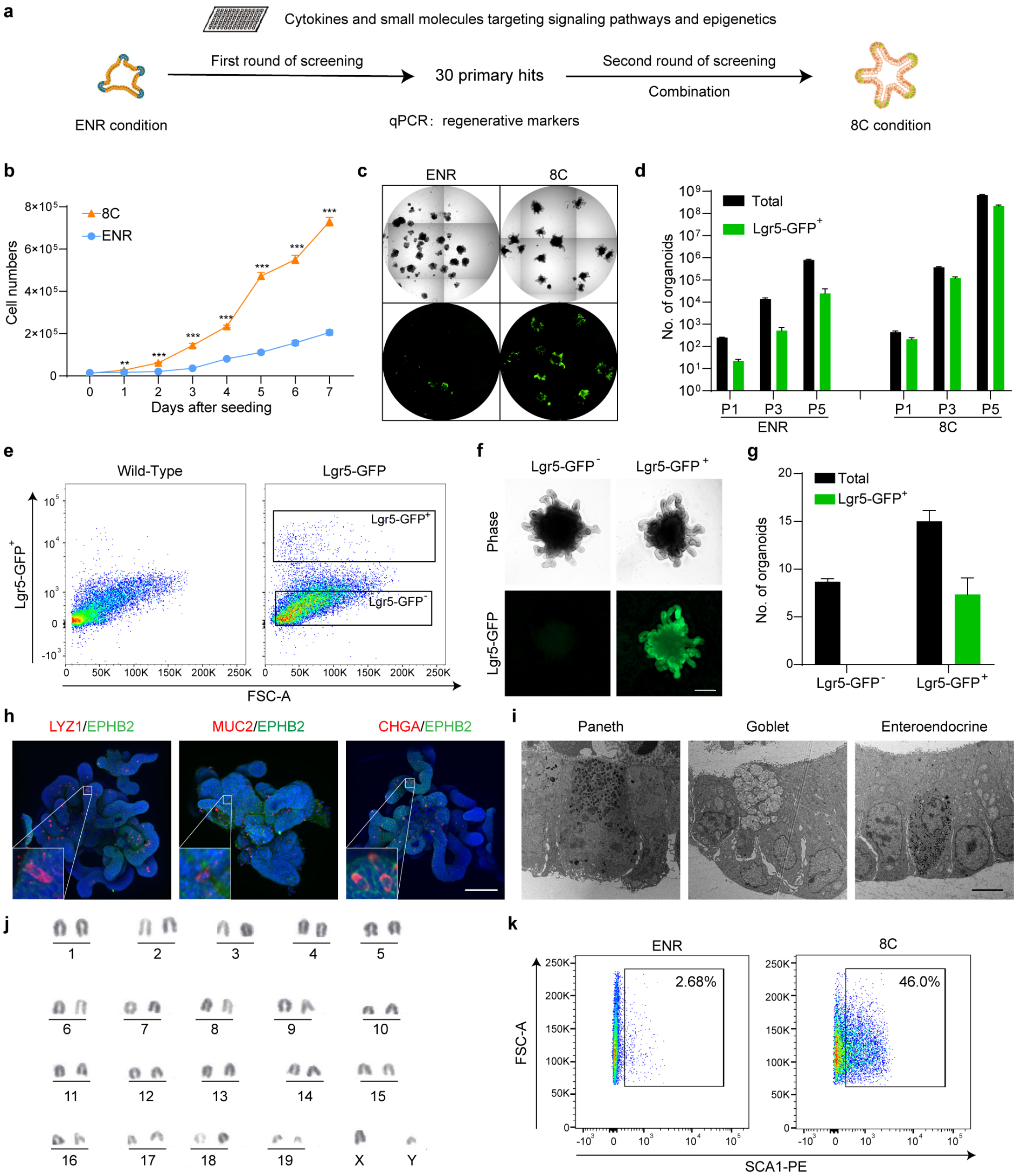

Supplementary information Fig. S1 Characterization of hyperplastic intestinal organoids

a, Scheme of screening strategy for inducing intestinal organoids with regenerative features.

b, Growth curves of organoids under the indicated conditions within 7 days (n = 4 wells). *P* values were determined by two-sided unpaired *t*-test.

c, Representative images of organoids cultured under the indicated conditions.

d, Quantification of total/Lgr5-GFP-expressing organoid number at different passages (n = 4 wells).

e, FACS sorting of freshly isolated single Lgr5-GFP$^+$ and Lgr5-GFP$^-$ cells. GFP$^+$ and GFP$^-$ single cell populations were collected respectively.

f, Representative images of organoids from single Lgr5-GFP$^+$ and Lgr5-GFP$^-$ cells cultured under the 8C condition. Scale bars, 100 μm.

g, Quantification of total/Lgr5-GFP-expressing organoid number from single Lgr5-GFP$^+$ and Lgr5-GFP$^-$ cells cultured under the 8C condition (n = 3 wells).

h, Immunofluorescence staining of representative marker genes expressed by Paneth (*Lyz1*), enteroendocrine (*Chga*) and goblet (*Muc2*) cells in organoids cultured in the 8C condition. Scale bars, 100 μm.

i, Detection of Paneth, enteroendocrine and goblet cells in organoids cultured in the 8C condition by transmission electron microscopy. Scale bars, 4 μm.

j, Metaphase spread of a cell cultured in the 8C condition over 140 d.

k, FACS analysis of SCA1$^+$ cells in different organoids shown in Fig. 1d.

***$P < 0.001$; **$P < 0.01$. All experiments were independently replicated at least twice with similar results.
