# Supplementary material for: Establishment of intestinal organoid cultures modeling injury-associated epithelial regeneration
Source: Cell Res. 2021 Jan 8;31(3):259–71. doi: 10.1038/s41422-020-00453-x (PMC8027647; doi:10.1038/s41422-020-00453-x)
Supplement: Supplementary file 2 — Supplementary Fig. S2 [file 41422_2020_453_MOESM2_ESM.pdf]

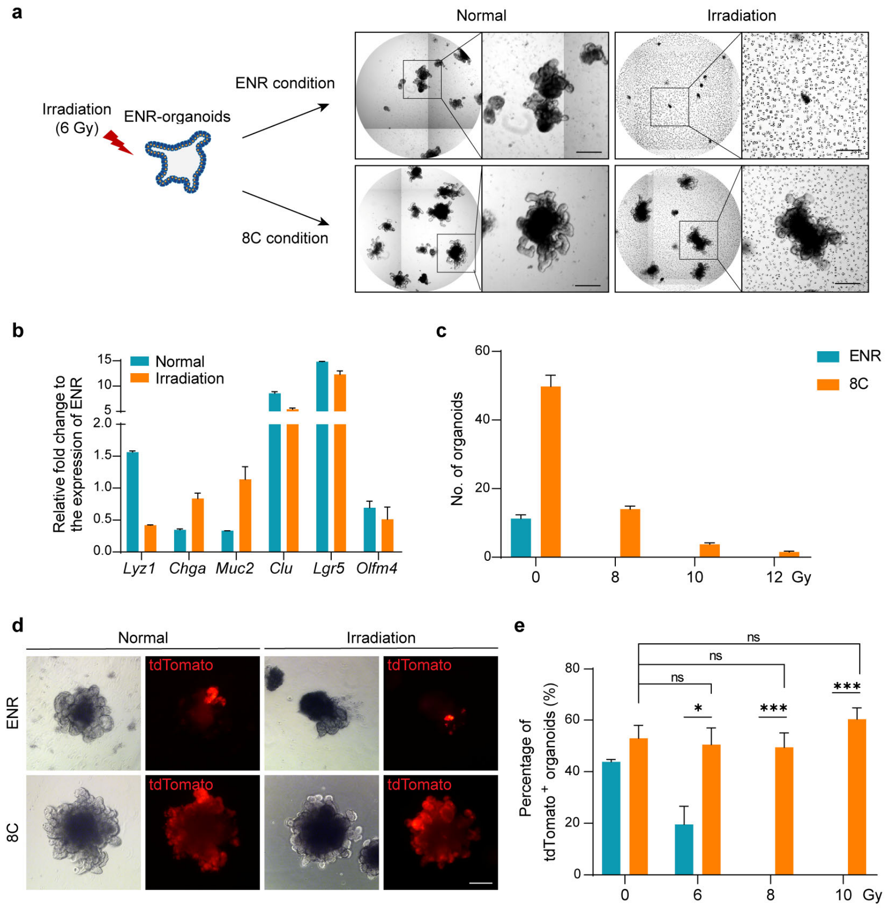

Supplementary information Fig. S2 Organoids cultured in the 8C condition survive in an *in vitro* irradiation model

a, Representative images of intestinal organoids cultured under the indicated conditions with or without 6 Gy γ-irradiation. Scale bars, 100 μm.

b, qPCR analyses of intestinal lineage markers in Hyper-organoids cultured under the indicated conditions (n = 2 wells).

c, Quantification of organoid number under the indicated culture conditions (n = 4 wells).

d, Representative images of intestinal organoids cultured under the indicated conditions with or without 6 Gy γ-irradiation using the lineage tracing system. Scale bars, 100 μm.

e, Quantification of tdTomato^{+} organoids under the indicated conditions (n = 4 wells).

P values were determined using one-way ANOVA.

***$P < 0.001$; **$P < 0.01$; *$P < 0.05$; ns, not significant ($P > 0.05$). All experiments were independently replicated at least twice with similar results.
