# Supplementary material for: Establishment of intestinal organoid cultures modeling injury-associated epithelial regeneration
Source: Cell Res. 2021 Jan 8;31(3):259–71. doi: 10.1038/s41422-020-00453-x (PMC8027647; doi:10.1038/s41422-020-00453-x)
Supplement: Supplementary file 3 — Supplementary Fig. S3 [file 41422_2020_453_MOESM3_ESM.pdf]

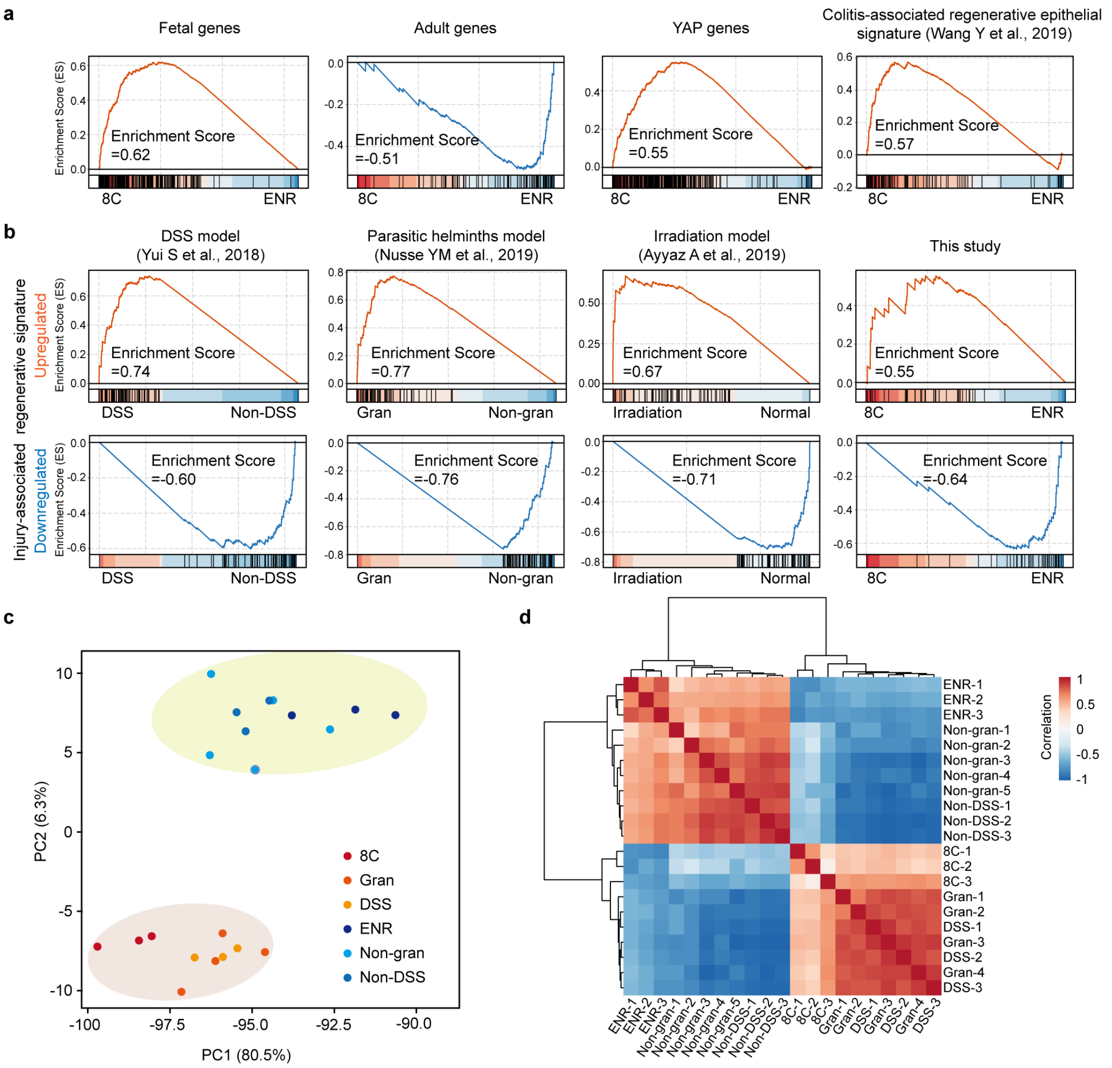

Supplementary information Fig. S3 Transcriptome analysis of Hyper-organoids

a, GSEA analysis showing enrichment of intestinal epithelial signature genes and colitis-associated regenerative genes in organoids cultured under the indicated conditions.

b, GSEA analysis showing enrichment of an injury-associated regenerative signature across different intestinal epithelium and organoids.

c, d, PCA analysis (c) and correlation matrix (d) of bulk RNA-seq data showing that Hyper-organoids resembled injury-associated epithelial cells more closely than ENR-organoids.
