# Supplementary material for: Establishment of intestinal organoid cultures modeling injury-associated epithelial regeneration
Source: Cell Res. 2021 Jan 8;31(3):259–71. doi: 10.1038/s41422-020-00453-x (PMC8027647; doi:10.1038/s41422-020-00453-x)
Supplement: Supplementary file 4 — Supplementary Fig. S4 [file 41422_2020_453_MOESM4_ESM.pdf]

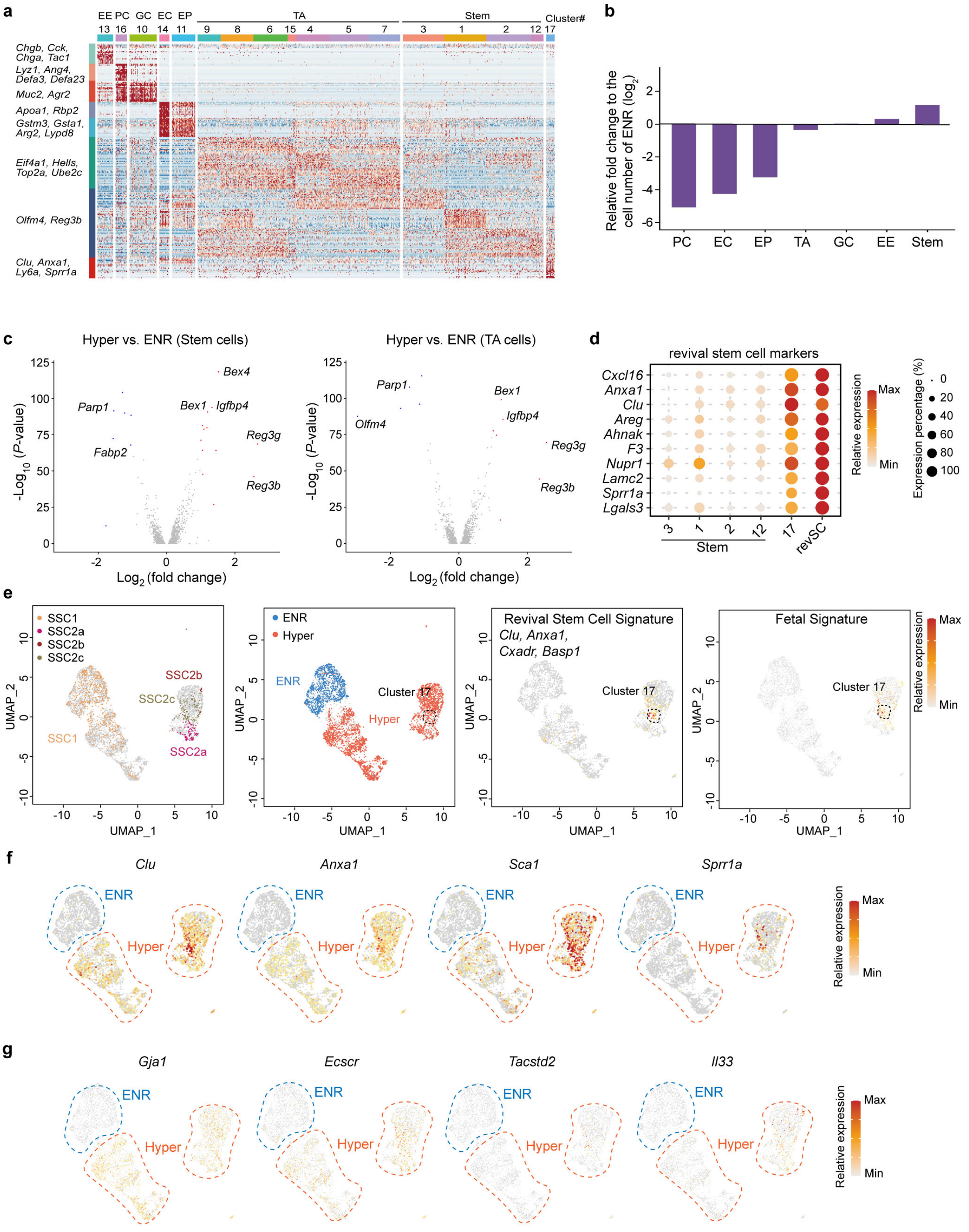

Supplementary information Fig. S4 Single-cell transcriptome analysis of Hyper-organoids

a, Expression of marker genes to identify distinct cell types in Hyper-organoids and ENR-organoids. Representative genes are shown on the left.

b, Comparison of lineage composition between Hyper-organoids and ENR-organoids.

c, Volcano plot displaying the results of differential gene-expression analysis performed in single stem cells (left) and single TA cells (right) in Hyper-organoids and ENR-organoids. The top differentially expressed genes (DEGs) are indicated.

d, Dot plot showing scaled expression of revival stem cell marker genes in clusters 1, 2, 3, 12, and 17 from this study and revival stem cells from an irradiation model.

e, Integrated analysis of stem cell clusters from different organoids *in vitro* and the irradiation model *in vivo*. UMAP visualizations of scRNA-seq data from clusters 1, 2, 3, 12, and 17 of organoids (middle), as well as SSC1, SSC2a, SSC2b, and SSC2c from the irradiation model (left). Analysis of cluster 17 showing expression of representative marker genes that distinguish SSC2c (middle) and the fetal (right) signature. Cells from different samples are also plotted in different colors as indicated.

f, g, The expression levels of revival stem cell (f) and fetal (g) marker genes in Hyper-organoids and ENR-organoids were overlaid on the UMAP shown in Fig. 2d. Hyper-organoids and ENR-organoids are separated by the orange and blue dotted line.
