# Supplementary material for: Establishment of intestinal organoid cultures modeling injury-associated epithelial regeneration
Source: Cell Res. 2021 Jan 8;31(3):259–71. doi: 10.1038/s41422-020-00453-x (PMC8027647; doi:10.1038/s41422-020-00453-x)
Supplement: Supplementary file 5 — Supplementary Fig. S5 [file 41422_2020_453_MOESM5_ESM.pdf]

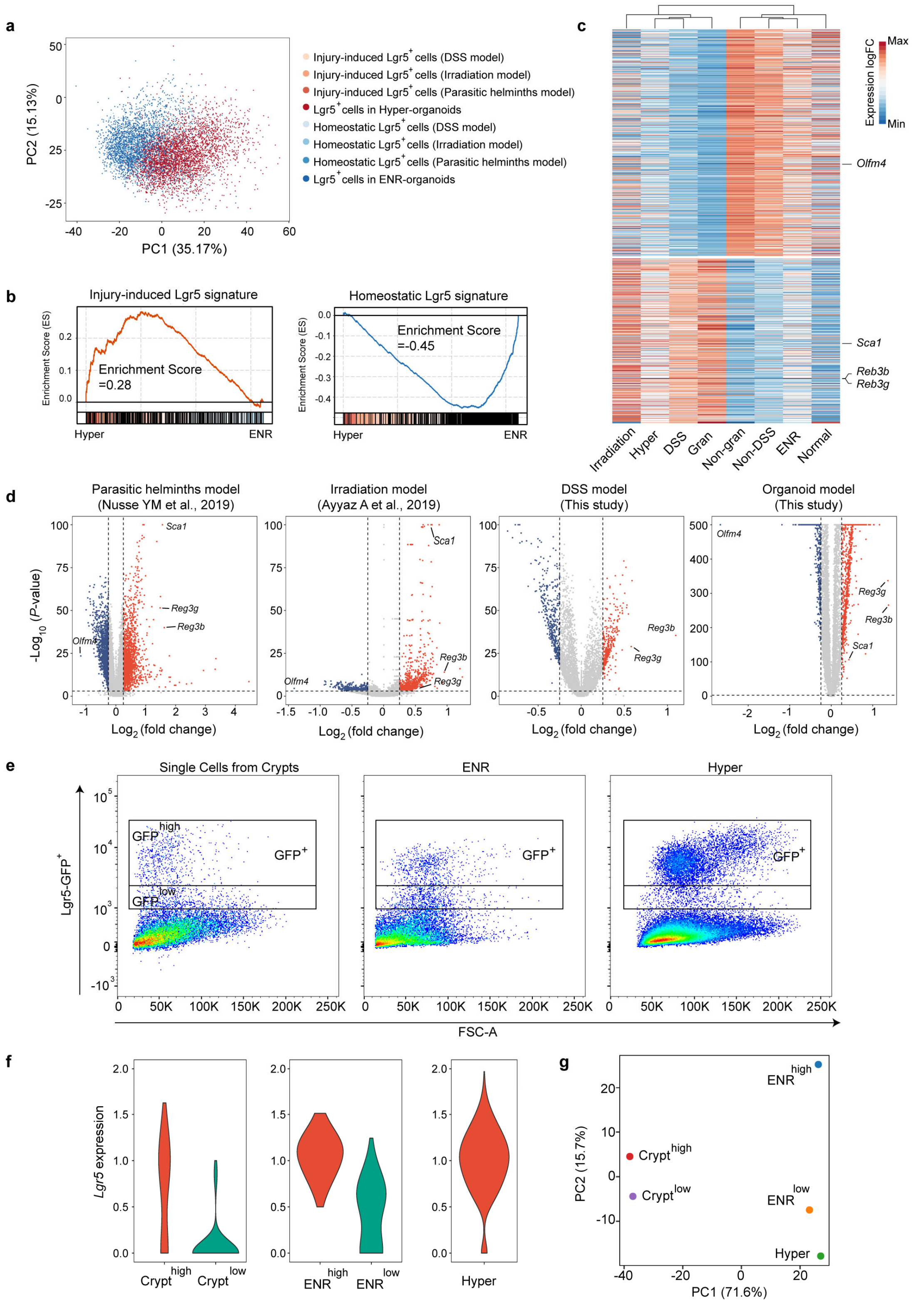

Supplementary information Fig. S5 Single-cell transcriptome analysis of injury-responsive and homeostatic Lgr5^{+} cells

a. PCA analysis of scRNA-seq data showing that Lgr5^{+} cells of Hyper-organoids

resembled injury-responsive Lgr5$^+$ cells *in vivo*.

b, GSEA analysis showing enrichment of injury-responsive Lgr5 signature and homeostatic Lgr5 signature in Lgr5$^+$ cells of ENR and Hyper-organoids.

c, Heatmap displaying the expression log fold change of an injury-responsive Lgr5 signature in different organoids and primary crypts from different intestinal injury models. The expression profile of organoids cultured in the ENR and 8C conditions were plotted. Representative genes are shown on the right. Gran and Non-gran indicate crypts overlying and adjacent to granulomas, respectively. DSS and Non-DSS indicate crypts from repairing epithelium during dextran sulfate sodium (DSS)-associated colitis, and normal epithelium without DSS treatment, respectively.

d, Volcano plot displaying the results of differential gene-expression analysis performed in injury-responsive Lgr5$^+$ cells and homeostatic Lgr5$^+$ cells. The top DEGs are indicated.

e, FACS analysis of single cells showed two distinct GFP^{high} and GFP^{low} populations, which represented Lgr5-high and Lgr5-low subsets, respectively.

f, Volcano plot displaying the *Lgr5* expression in single cells to define the Lgr5-high or Lgr5-low subsets.

g, PCA analysis of scRNA-seq data showing that Lgr5$^+$ cells in the Hyper-organoids were distinct from Lgr5-high and Lgr5-low cells in the ENR organoids.
