# Supplementary material for: Establishment of intestinal organoid cultures modeling injury-associated epithelial regeneration
Source: Cell Res. 2021 Jan 8;31(3):259–71. doi: 10.1038/s41422-020-00453-x (PMC8027647; doi:10.1038/s41422-020-00453-x)
Supplement: Supplementary file 6 — Supplementary Fig. S6 [file 41422_2020_453_MOESM6_ESM.pdf]

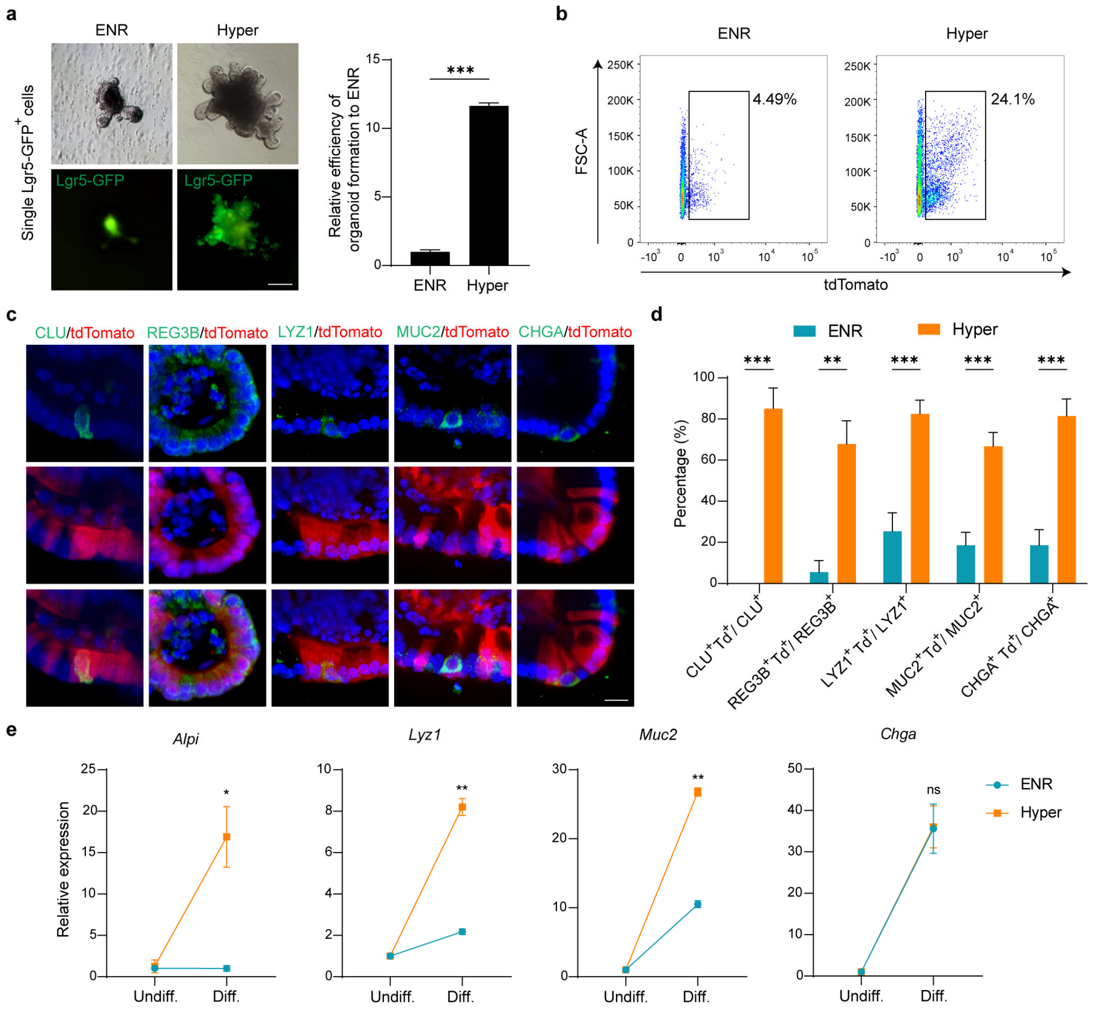

Supplementary information Fig. S6 Hyper-organoids contained functional Lgr5^{+} regenerative stem cells

a, Representative images and relative organoid-forming efficiency of single Lgr5^{+} cells from ENR and Hyper organoids.

b, FACS analysis of tdTomato^{+} cells in different organoids shown in Fig. 2f.

c, Immunofluorescence staining of regenerative and differentiated intestinal marker genes expressed in Hyper-organoids using the lineage tracing system. Scale bars, 10 μm.

d. Quantification of the proportion of cells expressing tdTomato and differentiated intestinal marker gene. *P* values were determined using one-way ANOVA.

e. qPCR analysis of differentiated intestinal gene expression in ENR and Hyper-organoids treated with differentiation medium for 3d or untreated.

***$P < 0.001$; **$P < 0.01$; *$P < 0.05$; ns, not significant ($P > 0.05$). All experiments were independently replicated at least twice with similar results.
