# Supplementary material for: Establishment of intestinal organoid cultures modeling injury-associated epithelial regeneration
Source: Cell Res. 2021 Jan 8;31(3):259–71. doi: 10.1038/s41422-020-00453-x (PMC8027647; doi:10.1038/s41422-020-00453-x)
Supplement: Supplementary file 7 — Supplementary Fig. S7 [file 41422_2020_453_MOESM7_ESM.pdf]

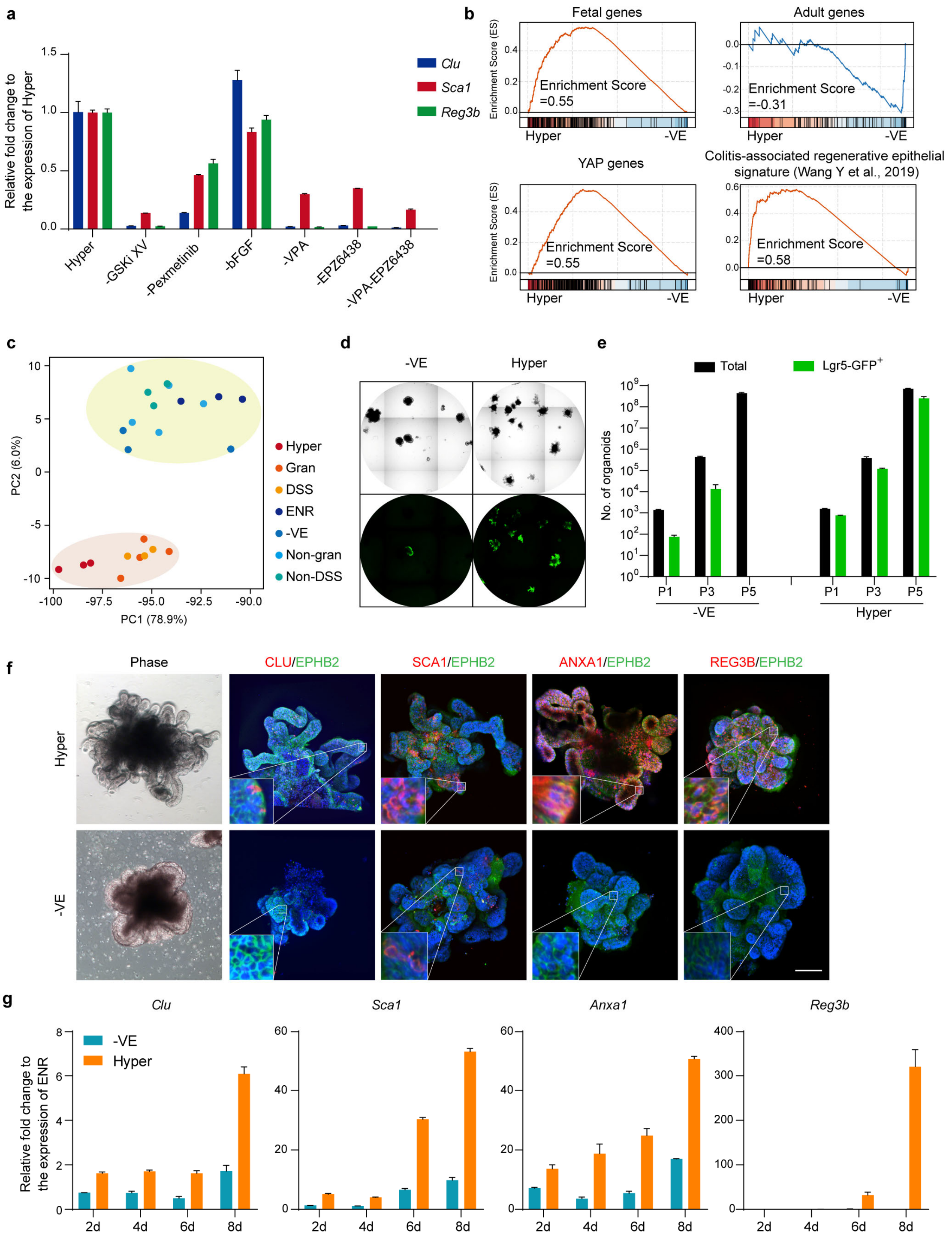

Supplementary information Fig. S7 VPA and EPZ6438 are critical for maintaining hyperplastic phenotype in intestinal organoids

a, qPCR analyses of regenerative markers in organoids cultured under the indicated conditions for 2 passages (n = 2 wells).

b, GSEA analysis for intestinal epithelial signature genes and colitis-associated regenerative genes in Hyper-organoids with and without VPA and EPZ6438.

c, PCA analysis showing that Hyper-organoids without VPA/EPZ6438 resembled ENR-organoids and homeostatic epithelial cells more closely than Hyper-organoids.

d, Representative images of Hyper-organoids with and without VPA/EPZ6438.

e, Quantification of total/Lgr5-GFP-expressing organoid number at different passages (n = 4 wells).

f, Immunofluorescence staining of CLU and fetal genes in Hyper-organoids with and without VPA/EPZ6438. Scale bars, 100 μm.

g, qPCR analysis of indicated gene expression in -VE and Hyper-organoids treated for different days.

***$P < 0.001$. The experiments in a, d, e and f were independently replicated at least twice with similar results.
