# Supplementary material for: Establishment of intestinal organoid cultures modeling injury-associated epithelial regeneration
Source: Cell Res. 2021 Jan 8;31(3):259–71. doi: 10.1038/s41422-020-00453-x (PMC8027647; doi:10.1038/s41422-020-00453-x)
Supplement: Supplementary file 8 — Supplementary Fig. S8 [file 41422_2020_453_MOESM8_ESM.pdf]

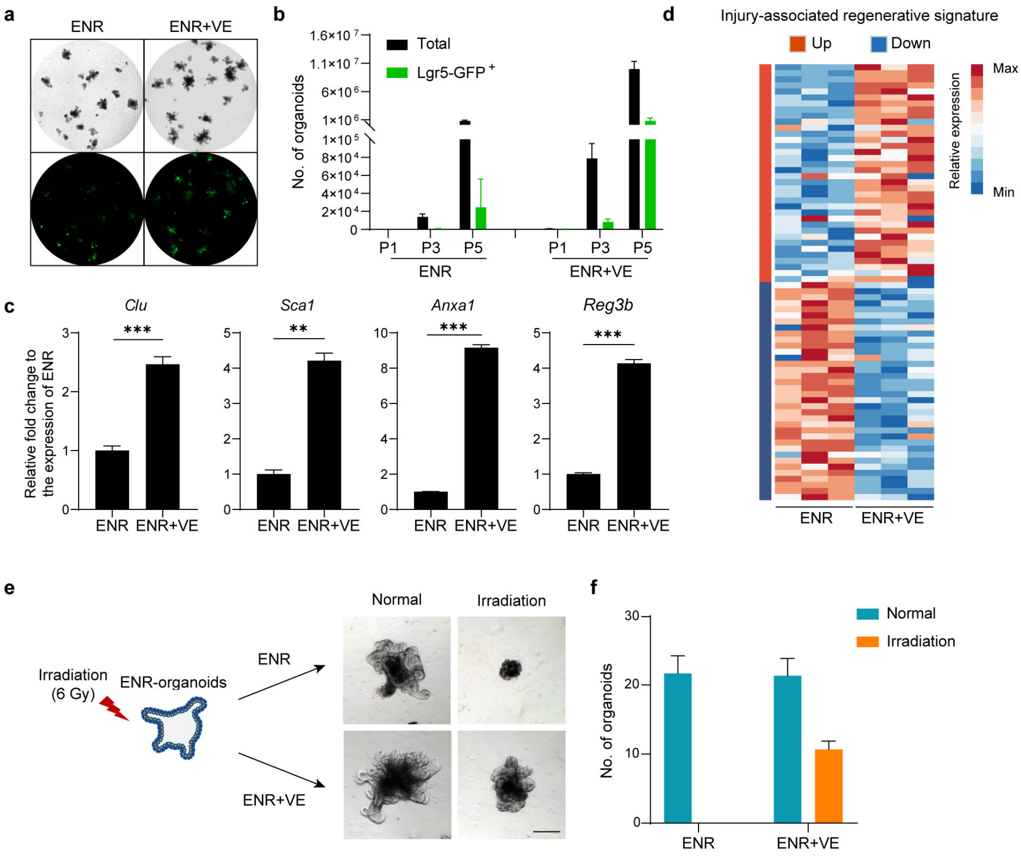

Supplementary information Fig. S8 VPA and EPZ6438 promote the regenerative response in ENR-organoids

a, Representative images of ENR-organoids with and without VPA/EPZ6438.

b, Quantification of total/Lgr5-GFP-expressing organoid number at different passages (n = 4 wells).

c, qPCR analyses of Clu and regenerative genes expression in organoids cultured under the indicated conditions (n = 2 wells). *P* values were determined by two-sided unpaired t-test.

d, Heatmap displaying the expression of an injury-induced regenerative signature in different organoids (n = 3 mice).

e, f, Representative images (e) and quantification of organoid number (f) cultured under

the indicated conditions with or without 6 Gy γ-irradiation. Scale bars, 100 μm. (n = 4 wells).

***$P < 0.001$; **$P < 0.01$; All experiments were independently replicated at least three times with similar results.
