# Supplementary material for: Establishment of intestinal organoid cultures modeling injury-associated epithelial regeneration
Source: Cell Res. 2021 Jan 8;31(3):259–71. doi: 10.1038/s41422-020-00453-x (PMC8027647; doi:10.1038/s41422-020-00453-x)
Supplement: Supplementary file 9 — Supplementary Fig. S9 [file 41422_2020_453_MOESM9_ESM.pdf]

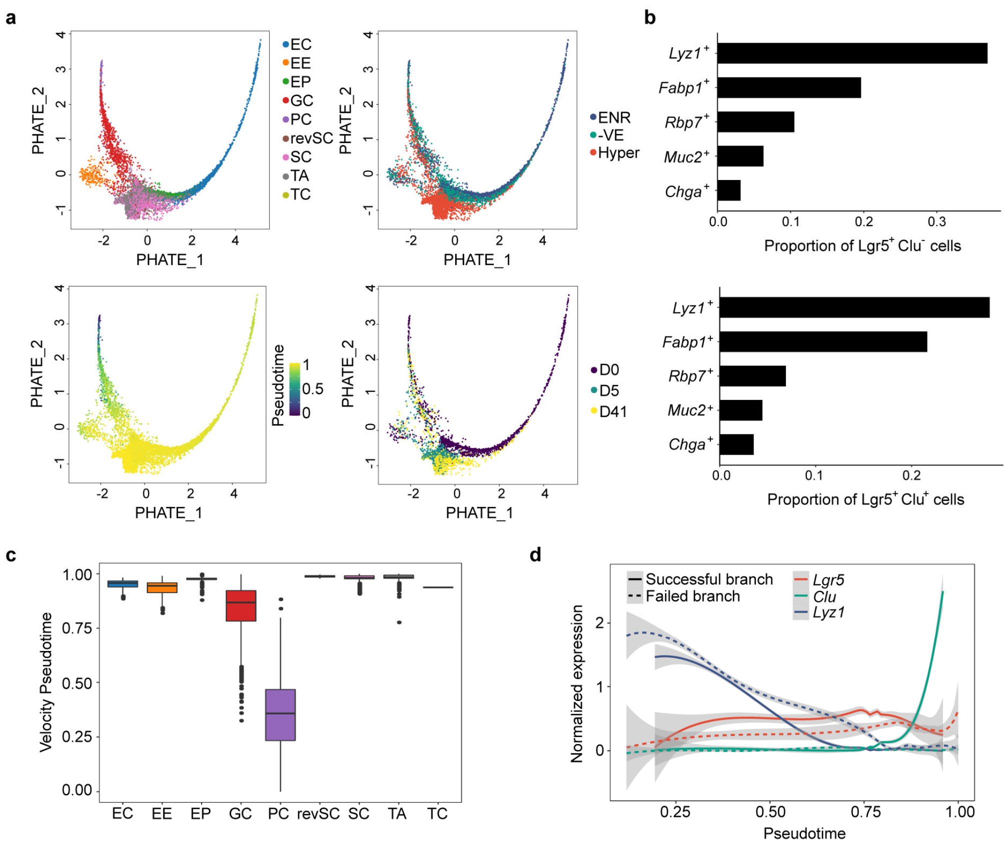

Supplementary information Fig. S9 Reconstruction of regenerative trajectory in Hyper-organoids in a pseudotime manner

a, Trajectory reconstruction of single cells from Hyper-organoids with and without VPA/EPZ6438 cultured for 5 days and 41 days. Cells colored by cell types overlaid by RNA velocity result (top left), and pseudo time calculated by RNA velocity (bottom left). Cells from distinct samples are plotted in different colors as indicated.

b, Percentage of cells co-expressing differentiation and stem cell markers from Hyper-organoids.

c, Boxplot showing pseudo time disperation of all cell types.

d, Expression dynamics of *Lgr5*, *Clu* and *Lyz1* was cataloged in a pseudotime manner shown as full lines (successful reprogramming), and dotted lines (failed reprogramming). Thick lines indicate the average gene expression patterns in each

branch.
