# Supplementary material for: Establishment of intestinal organoid cultures modeling injury-associated epithelial regeneration
Source: Cell Res. 2021 Jan 8;31(3):259–71. doi: 10.1038/s41422-020-00453-x (PMC8027647; doi:10.1038/s41422-020-00453-x)
Supplement: Supplementary file 10 — Supplementary Fig. S10 [file 41422_2020_453_MOESM10_ESM.pdf]

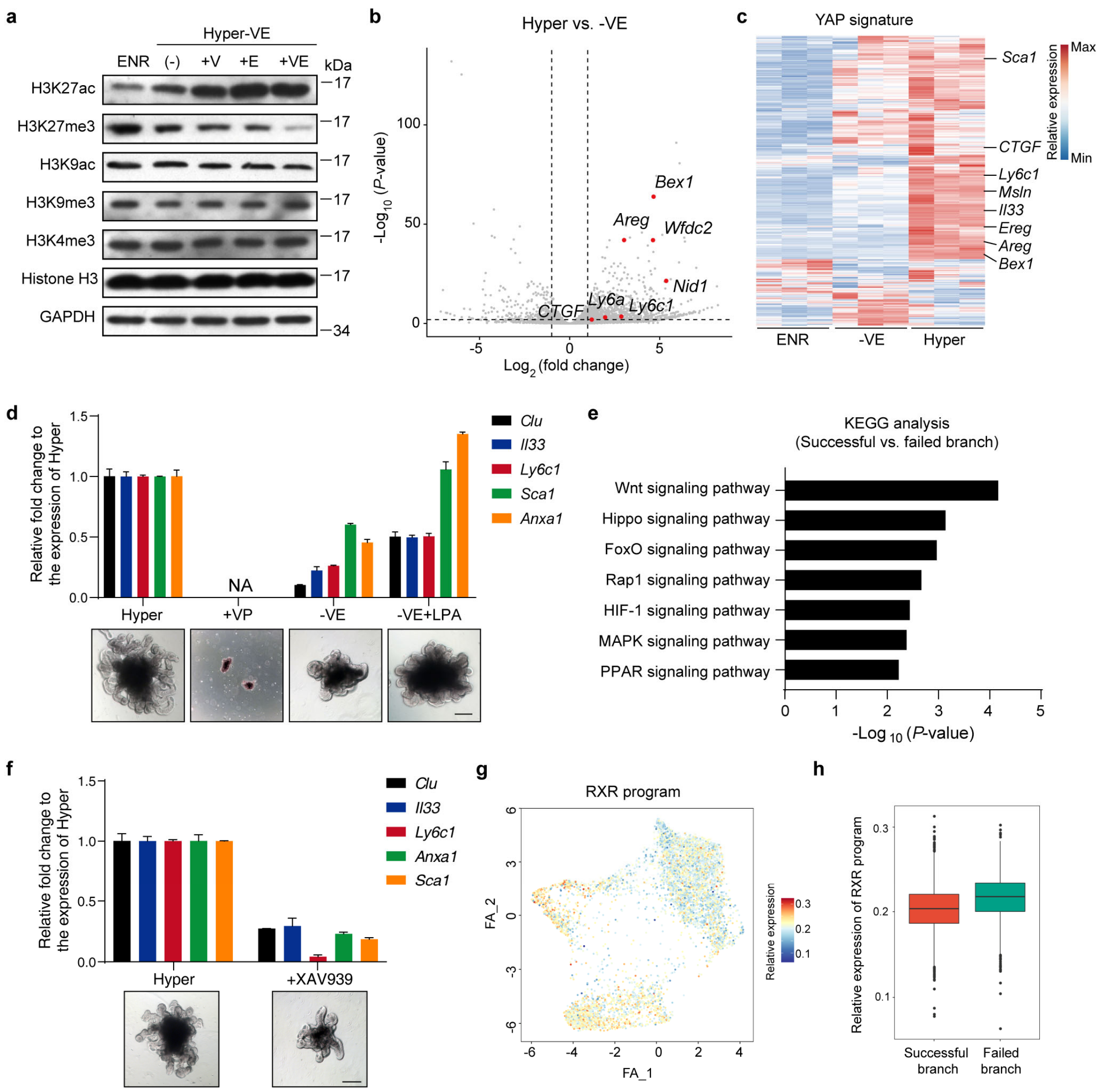

Supplementary information Fig. S10 Regulation of YAP plays a role in the VPA-and-EPZ6438-driven regenerative response *in vitro*

a, Western blot analysis of different histone marks in organoids cultured under different conditions using the indicated antibodies.

b, Volcano plot displaying the results of differential gene-expression analysis performed in bulk epithelial cells from Hyper-organoids with and without VPA/EPZ6438. YAP target genes are indicated.

c, Heatmap displaying the expression of YAP signature in different organoids (n = 3

mice).

d, qPCR analyses of regenerative genes in organoids cultured under the indicated conditions (n = 2 wells). The typical morphology of intestinal organoids cultured under the indicated conditions is shown below. Scale bars, 100 μm.

e, KEGG analysis showed the enrichment of signaling pathways in successful branch vs. failed branch during the regenerative trajectory.

f, qPCR analyses of regenerative genes in organoids cultured under the indicated conditions (n = 2 wells). The typical morphology of intestinal organoids cultured under the indicated conditions is shown below. Scale bars, 100 μm.

g, Expression of the genes containing RXR motifs in the promoter was overlaid on the regenerative trajectory from Hyper-organoids with and without VPA/EPZ6438 shown in Fig. 3f.

h, Box plots displaying expression of the genes containing RXR motifs in the promoter in successful and failed branch.

The experiments in a and d were independently replicated at least twice with similar results.
