# Supplementary material for: Establishment of intestinal organoid cultures modeling injury-associated epithelial regeneration
Source: Cell Res. 2021 Jan 8;31(3):259–71. doi: 10.1038/s41422-020-00453-x (PMC8027647; doi:10.1038/s41422-020-00453-x)
Supplement: Supplementary file 11 — Supplementary Fig. S11 [file 41422_2020_453_MOESM11_ESM.pdf]

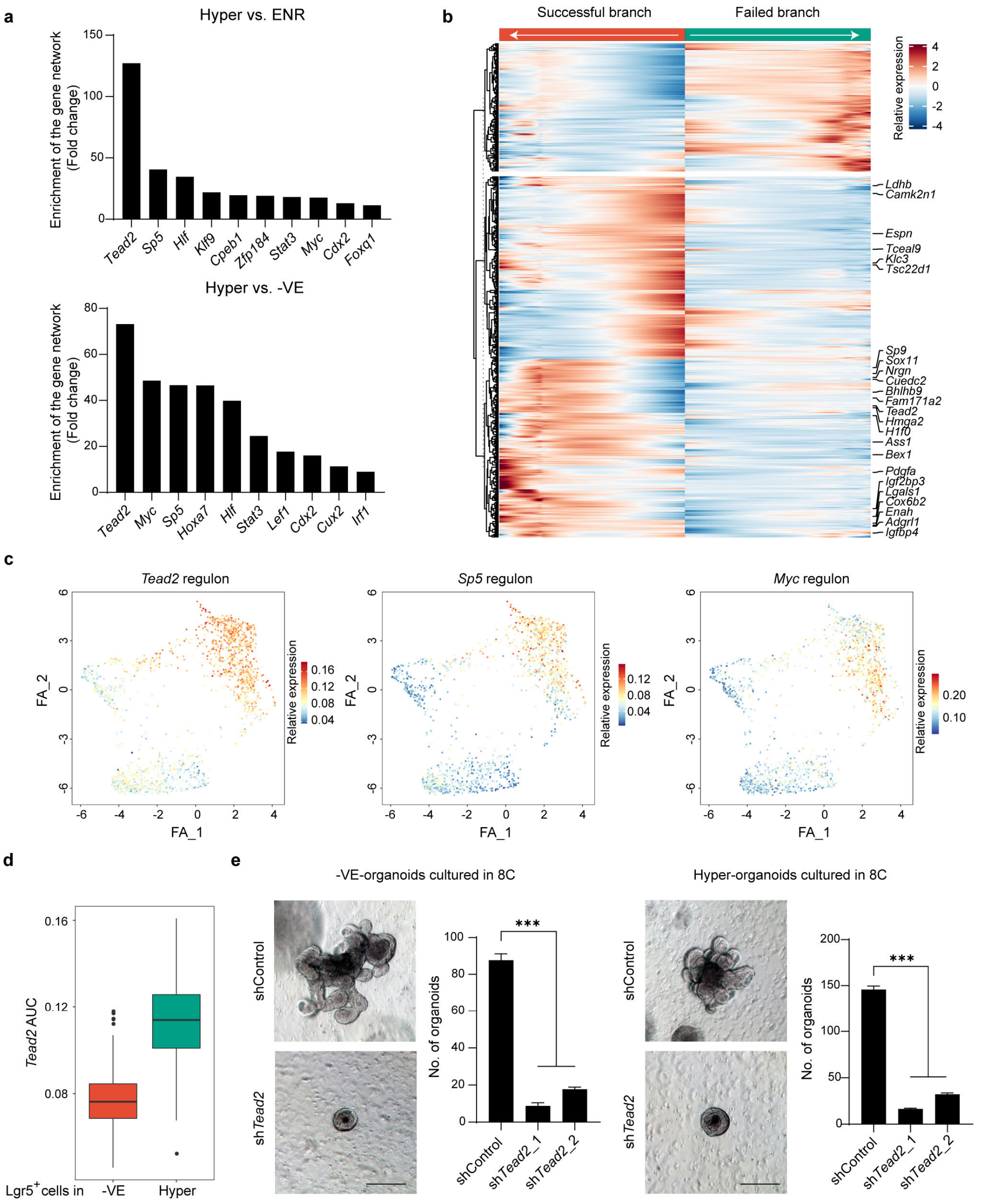

Supplementary information Fig. S11 SCENIC analysis reveals the gene regulatory network of Hyper-organoids

a, Enrichment of the gene network analyzed by SCENIC in all single cells from Hyper-organoids compared with ENR-organoids (top) and Hyper-organoids without VPA/EPZ6438 (bottom).

b, Gene expression heatmap of 500 differentially expressed genes between two branches in a pseudo-temporal order. Successful and failed regenerative trajectories (including pre-branch) are shown on the left and right, respectively. Representative genes of *Tead2* regulon are shown on the right.

c, ForceAtlas embedding plot displaying *Tead2*, *Sp5* and *Myc* regulons (red dots, active; blue dots, inactive) as well as their intensity distributions from Hyper-organoids with and without VPA/EPZ6438 shown in Fig. 3f.

d, Box plots displaying *Tead2* regulon activity measured by area under the curve (AUC) of Lgr5^{+} cell in –VE and Hyper organoids.

e, Representative images and quantification of total organoid number cultured under the indicated conditions after the knockdowns with the indicated shRNA. –VE-organoids (left) or Hyper-organoids (right) were cultured in the 8C condition for 10 days. Scale bars, 100 µm.
