# Supplementary material for: Establishment of intestinal organoid cultures modeling injury-associated epithelial regeneration
Source: Cell Res. 2021 Jan 8;31(3):259–71. doi: 10.1038/s41422-020-00453-x (PMC8027647; doi:10.1038/s41422-020-00453-x)
Supplement: Supplementary file 12 — Supplementary Fig. S12 [file 41422_2020_453_MOESM12_ESM.pdf]

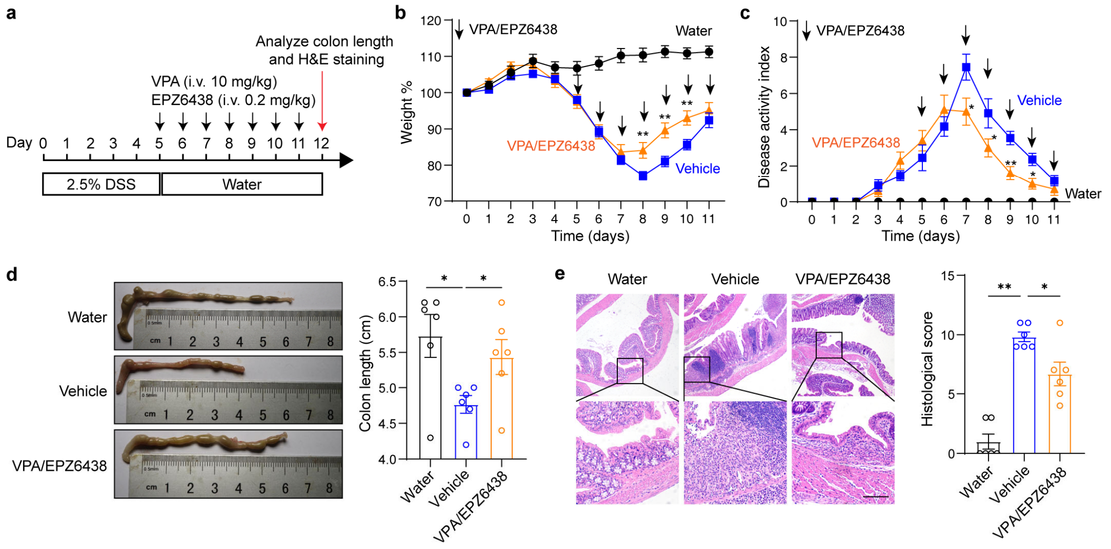

Supplementary information Fig. S12 Combined treatment with VPA and EPZ6438 alleviates DSS-associated colitis

a, Scheme of the therapeutic treatment with VPA/EPZ6438 on DSS-associated colitis in C57BL/6 mice.

b, c, Body weight **(b)** and disease activity index **(c)** of mice that received regular drinking water alone (Water group, black dotted line), or 2.5% DSS combined with vehicle treatment (Vehicle group, blue dotted line), or 2.5% DSS combined with VPA and EPZ6438 treatment (VPA/EPZ6438 group, orange dotted line) (n = 12 per group). *P* values were determined using one-way ANOVA and two-tailed Mann–Whitney test for body weight and disease activity index, respectively.

d, Colon length at day 12 after DSS induction (n = 6 per group). *P* values were determined using one-way ANOVA.

e, Representative H&E staining of colon sections, and corresponding histological scores at day 12 after DSS induction (n = 6 per group). Scale bars, 50 µm. *P* values were determined by two-tailed Mann–Whitney test.

***$P < 0.001$; **$P < 0.01$; *$P < 0.05$. Experiments in c, d and e were independently replicated at least twice with similar results.
