# Supplementary material for: Establishment of intestinal organoid cultures modeling injury-associated epithelial regeneration
Source: Cell Res. 2021 Jan 8;31(3):259–71. doi: 10.1038/s41422-020-00453-x (PMC8027647; doi:10.1038/s41422-020-00453-x)
Supplement: Supplementary file 13 — Supplementary Table S1 [file 41422_2020_453_MOESM13_ESM.pdf]

Table S1. Gene set for the injury-associated regenerative signature

Upregulated genes

AW112010	Duoxa2	Hs3st1	Mecom	Ripk3	Stat2	Ywhag
Batf2	Egln3	Icam1	Mst1r	Rnf213	Sulf2	Zbp1
C3	Eif4e3	Ido1	Myo9b	S100a11	Tap1	Zc3h7a
Capg	Epha2	Ier3	Pitpnm1	Sbno2	Tgm2	
Cd74	Gadd45g	Ifi47	Pkp4	Slc7a11	Tmc6	
Cdk11b	Gbp7	Igtp	Plec	Socs1	Tnfrsf1b	
Cxcl2	H2-Ab1	Il4ra	Plk2	Socs3	Trafd1	
D16Ertd472e	H2-Q7	Lpcat4	Reg3b	Sprr1a	Trim15	
Ddah1	Hk2	Lrg1	Reg3g	Srgn	Trim40	
Duox2	Hmox1	Ly6a	Rhof	Stat1	Ubd	

Downregulated genes

Aadac	Bphl	Chpt1	Gas6	Lgr5	Sult1d1
Abat	Car4	Clps	Gde1	Maoa	Thra
Adh1	Cat	Cmbl	Gng11	Maob	Ugt2b35
Adtrp	Ccl28	Coq8a	Gstm3	Me2	Vdr
Akr1c14	Ccl6	Cth	Gstm7	Mme	
Aldh1a1	Ces1e	Cyp2c68	H2afv	Naxe	
Aoc1	Ces1f	D630039A03	Hadh	Oat	
Aqp11	Ces2a	Ddc	Hsd11b2	Otc	
Bche	Ces2c	Fahd1	Itln1	Pccb	
Bco2	Ces2g	Fmo4	Khk	Scin	
