# Supplementary material for: Establishment of intestinal organoid cultures modeling injury-associated epithelial regeneration
Source: Cell Res. 2021 Jan 8;31(3):259–71. doi: 10.1038/s41422-020-00453-x (PMC8027647; doi:10.1038/s41422-020-00453-x)
Supplement: Supplementary file 14 — Supplementary Table S2 [file 41422_2020_453_MOESM14_ESM.pdf]

Table S2 Primers used in this study

RT-qPCR primers

Gene	Forward	Reverse
Clu	ACAATCCTGCCCAGGCTAAC	CAGGGATGAGGTGTTGAGCA
Sca1	GATGGACACTTCTCACACTACA	GCAGGTAATTGATGGGCAAGA
Anxa1	CTGACCAGCAGGAGCTTTC	TGGCTTCGTACAGCTTCTCG
Reg3b	CCTTAGACCGTGCTTTCTGTG	GTCCATGATGCTCTTCAAGACA
