# Supplementary material for: Establishment of intestinal organoid cultures modeling injury-associated epithelial regeneration
Source: Cell Res. 2021 Jan 8;31(3):259–71. doi: 10.1038/s41422-020-00453-x (PMC8027647; doi:10.1038/s41422-020-00453-x)
Supplement: Supplementary file 15 — Supplementary Table S3 [file 41422_2020_453_MOESM15_ESM.pdf]

Table S3 scRNA-Seq sample quality data

Sample	Origin	Passage number	Symbol	Cells Number After QC	Average Genes Number	Average UMI Counts
Hyper-organoids	ENR-organoids	P1	S23	2812	3157	49254
-VE-organoids		P1	S22	2601	3213	52103
ENR-organoids		P2	S21	2189	3149	46998
Hyper-organoids	ENR-organoids	P6	S63	3523	3165	40535
-VE-organoids		P6	S62	4293	3205	41375
ENR-organoids		P7	S61	2601	3213	48014
Hyper-organoids	Primary crypts	P3	SC3	1983	3170	47158
ENR-organoids		P3	SC1	2348	3214	39382
Colonic crypts from DSS treated mice			D2	1933	3247	16760
Colonic crypts from normal mice			W4	1132	3340	17585
