# Supplementary material for: Establishment of intestinal organoid cultures modeling injury-associated epithelial regeneration
Source: Cell Res. 2021 Jan 8;31(3):259–71. doi: 10.1038/s41422-020-00453-x (PMC8027647; doi:10.1038/s41422-020-00453-x)
Supplement: Supplementary file 16 — Supplementary Table S4 [file 41422_2020_453_MOESM16_ESM.pdf]

Table S4 REAGENT or RESOURCE

REAGENT or RESOURCE	SOURCE	IDENTIFIER
Antibodies		
Anti-Clusterin antibody	Abcam	ab69644
Mouse Sca-1/Ly6 Antibody	R&D	MAB1226
Annexin A1 Polyclonal Antibody	Invitrogen	PA5-27315
Mouse Reg3B Antibody	R&D	MAB5110-SP
Anti-MUC2 antibody	Abcam	ab11197
Anti-Lysozyme antibody	Abcam	ab36362
Chr-A Antibody (C-12)	Santa Cruz Biotechnology	sc-393941
Human/Mouse EphB2 Antibody	R&D	AF467
PE Rat Anti-Mouse Ly-6A/E	BD	553108
anti-H3K9me3	Millipore	07- 449
anti-H3K9ac	Abcam	ab4441
anti-H3K27me3	Abcam	ab6002
anti-H3K27ac	Abcam	ab4729
anti-H3K4me3	Abcam	ab8580
anti-GAPDH	Proteintech	60004-1-Ig
anti-Histone-H3	Proteintech	17168-1-AP
Alexa Fluor ® 647 AffiniPure Donkey Anti-Goat IgG (H+L)	Jackson ImmunoResearch Laboratories	705-605-147
Cy ™ 3 AffiniPure Donkey Anti-Rabbit IgG (H+L)	Jackson ImmunoResearch Laboratories	711-165-152
Cy ™ 3 AffiniPure Donkey Anti-Mouse IgG (H+L)	Jackson ImmunoResearch Laboratories	715-165-150
Chemicals, Peptides, and Recombinant Proteins		
LDN-193189	Selleck	S2618
GSK-3 Inhibitor XV	CALBIOCHEM	361558
Pexmetinib (ARRY-614)	Selleck	S7799
Valproic acid sodium salt (VPA)	Sgima	P4543
Tazemetostat (EPZ-6438)	Selleck	S7128
Verteporfin (VP)	Selleck	S1786
LPA	Cayman	62215
Recombinant Murine EGF	PeproTech	315-09
Recombinant Murine Noggin	PeproTech	250-38
Recombinant Human R-Spondin-1	PeproTech	120-38
FGF2 (NM_002006) Human Recombinant Protein	Origene	TP750002
